# Repression of chimeric transcripts emanating from endogenous retrotransposons by a sequence-specific transcription factor

**DOI:** 10.1186/gb-2014-15-4-r58

**Published:** 2014-04-30

**Authors:** Ka Sin Mak, Jon Burdach, Laura J Norton, Richard CM Pearson, Merlin Crossley, Alister PW Funnell

**Affiliations:** 1 School of Biotechnology and Biomolecular Sciences, University of New South Wales, Kensington NSW 2052, Australia

**Keywords:** Retrotransposons, Chimeric transcripts, KLF1, KLF3, KLFs, Gene regulation, Erythroid, Transcription factors

## Abstract

**Background:**

Retroviral elements are pervasively transcribed and dynamically regulated during development. While multiple histone- and DNA-modifying enzymes have broadly been associated with their global silencing, little is known about how the many diverse retroviral families are each selectively recognized.

**Results:**

Here we show that the zinc finger protein Krüppel-like Factor 3 (KLF3) specifically silences transcription from the *ORR1A0* long terminal repeat in murine fetal and adult erythroid cells. In the absence of KLF3, we detect widespread transcription from *ORR1A0* elements driven by the master erythroid regulator KLF1. In several instances these aberrant transcripts are spliced to downstream genic exons. One such chimeric transcript produces a novel, dominant negative isoform of PU.1 that can induce erythroid differentiation.

**Conclusions:**

We propose that KLF3 ensures the integrity of the murine erythroid transcriptome through the selective repression of a particular retroelement and is likely one of multiple sequence-specific factors that cooperate to achieve global silencing.

## Background

Transposable elements (TEs) are mobile segments of DNA that have integrated and spread in eukaryotic genomes. They constitute a substantial portion of the mouse and human genomes (approximately 39% and 46%, respectively [1]), and have been a major driving force in evolution [2]. In mammals, the vast majority of TEs are retrotransposons: genetic elements that have integrated into host DNA following reverse transcription of an RNA template. Broadly, retrotransposons fall into two categories: those that contain long terminal repeats (LTRs) and are termed endogenous retroviruses (ERVs); and those that lack LTRs, namely, long and short interspersed elements (LINES and SINES).

The expansion of TEs has played an important role in shaping eukaryotic genomes, in part by enabling genomic shuffling by non-allelic homologous recombination but also by their capacity to remodel gene regulatory networks [3-7]. Retroviral LTRs, for instance, harbor numerous, functional regulatory elements required for the initiation and control of transcription and can thus profoundly alter the expression of proximal genes [8,9]. Furthermore, because the many distinct classes of retrovirus differ in the regulatory sequences contained within their LTRs, they have proven highly versatile in rewiring diverse transcriptional programs. Indeed, throughout mammalian evolution, the spread and proliferation of retroelements have redistributed binding sites for a number of transcriptional regulators including the pluripotency factors OCT4 and NANOG [5], the insulator protein CTCF [4-6], the neural repressor NRSF/REST [10], the tumour suppressor p53 [11], and others [12]. Analogously, expansion of the *MER20* and *RLTR13D5* transposable elements, which promote endometrial and trophoblast expression, have been postulated to have enabled the placental transcription of genes critical to the evolutionary development of pregnancy [13,14].

Not only do retrotransposons provide regulatory modules that influence nearby genes, they can also directly provide promoters that dictate transcriptional initiation. A notable example of this is the murine *Agouti viable yellow* (*A*^*vy*^) allele, in which an upstream intra-cisternal A particle (IAP) retrotransposon functions as a constitutively active promoter that drives ectopic expression of Agouti, resulting in yellow fur, obesity, and increased susceptibility to tumorigenesis [15,16]. The prevalence of this phenomenon, whereby retrotransposons serve as alternative promoters, has recently been revealed following the advent of high-throughput RNA sequencing and shown to occur primarily in embryonic cells but also to some extent in adult tissues [17]. During early embryonic development in particular, up to 20% of the transcriptome has been shown to initiate from within retrotransposons [17,18]. These retroelements frequently function as alternative promoters and show a propensity for tissue-specific activity, more so in fact than non-retrotransposon promoters [17]. In many instances, these retrotransposons have been co-opted by the host by exonization and they are transcribed and spliced to downstream genic exons [17,19,20]. The resulting chimeric transcripts thus potentially encode isoform variants with spatio- or temporally-restricted expression profiles [21]. Indeed, a recent study of the *Drosophila melanogaster* transcriptome has revealed that several hundred LTR retrotransposons serve as promoters of annotated genes throughout development, exhibiting specific expression profiles depending on the different regulatory modules they carry [22].

However, while there are many reported instances of TEs being co-opted by the host for various biological functions, genomic integration of TEs can also be deleterious [23-25]. For instance, Hodgkin’s lymphoma has been shown to arise from aberrant transcription of the *colony-stimulating factor 1 receptor* (*CSF1R*) gene driven by an internal LTR element known as *THE1B*[26].

Accordingly, higher eukaryotes have developed numerous defence mechanisms to silence TEs, typically involving DNA methylation and/or histone modification [27-29]. This silencing largely occurs early in embryonic development and is dependent on epigenetic modifiers including: DNA methyltransferases (DNMTs) [27,30]; histone modifying enzymes such as the demethylase LSD1/KDM1A, the deacetylase HDAC1, and the methyltransferases SETDB1 and G9A [31-35]; and Polycomb Group proteins [36]. Ablation of these factors in embryonic stem cells results in widespread de-repression of retrotransposon-derived transcripts.

However, while silencing of retroelements is broadly carried out by these epigenetic modifiers, little is known about the underlying mechanisms by which the diverse classes of retroelements are each specifically recognized [28]. Indeed the lack of sequence similarity between unrelated retroviral families suggests the existence of multiple recognition factors that participate in the silencing of retroelements. An accumulating body of evidence has pointed towards the possible role of DNA binding, tandem zinc finger proteins in providing this specificity. Thomas and Schneider have proposed a model of co-evolution between retroelements and C_2_H_2_ zinc finger proteins based on striking correlations of their expansion throughout vertebrate genomes [37]. This model followed from the discovery that the Krüppel-associated box (KRAB)-zinc finger protein ZFP809 binds and represses a large number of retroelements in mouse embryonic stem cells [38]. ZFP809 achieves this through the recruitment of the corepressor TRIM28 (also known as KRAB-associated protein 1, KAP1). TRIM28 in turn silences ERVs through SETDB1 mediated trimethylation of H3K9 [31,39,40].

The Krüppel-like factors (KLFs) are a family of DNA binding, zinc finger transcription factors [41]. They lack a KRAB domain and are characterized by a set of three tandem C_2_H_2_ zinc fingers at their C-termini that confer specificity towards CACCC-like and GC-rich sequences in regulatory elements [42]. While the DNA binding domain is highly conserved within the family, the N-terminal regulatory domains vary considerably such that the different KLFs recruit an assortment of co-regulators to activate or repress genes [43].

The founding member of the family, KLF1, is an erythroid-specific transcriptional activator that drives the expression of genes required for red blood cell maturation [44]. One such gene is that encoding the related family member KLF3 [45]. KLF3 and KLF1 recognize similar sequences of DNA that adhere to the consensus 5′-NCN CNC CCN-3′ [42,46]. However, unlike KLF1, KLF3 is a transcriptional repressor that recruits the co-repressor C-terminal binding protein (CTBP) [47]. CTBP forms part of a large repressor complex that includes the histone deacetylases HDAC1 and HDAC2, the histone methyltransferases EHMT1 and G9A/EHMT2, and the lysine-specific demethylase LSD1/KDM1A [48]. KLF1 and KLF3 exhibit opposing activities at a number of genes in erythroid cells and serve to fine-tune their expression during erythropoiesis [49,50]. Accordingly, loss of either factor disrupts this balance. *Klf1* null mice die of severe anemia *in utero* while mice lacking KLF3, though viable, exhibit erythroid defects in both fetal and adult tissues [49,51].

Here, we have further explored the interplay between KLF1 and KLF3 in regulating the erythroid transcriptome. We find that KLF1 activates, while KLF3 represses, transcription from a specific family of LTR elements known as *ORR1A0*. Ablation of KLF3 results in widespread, de-repressed transcription from these LTRs in erythroid cells. Because the *ORR1A0* element contains an intact splice donor site, these transcripts are spliced to exons of the genes in which they reside. We show that for the *spleen focus forming virus proviral integration 1* (*Sfpi1*) gene, an *ORR1A0*-driven transcript is translated into a truncated variant of PU.1 which exhibits dominant negative activity and can functionally promote erythroid differentiation. These results suggest that KLF3 ensures normal murine erythropoiesis by preventing aberrant, chimeric transcription driven from *ORR1A0* LTRs by KLF1.

## Results

### Increased expression of downstream *Pu.1* exons in erythroid cells in the absence of KLF3

We recently identified a number of KLF3 target genes via microarray analysis of *Klf3*^−/−^ TER119^+^ (erythroid) fetal liver cells at embryonic day E14.5 [49]. These genes were predominantly de-repressed in *Klf3* null tissue, consistent with KLF3 being a repressor of transcription. One of the most highly de-repressed genes was that encoding the key hematopoietic regulator PU.1/SFPI1, hereafter referred to as PU.1.

**Figure 1:**
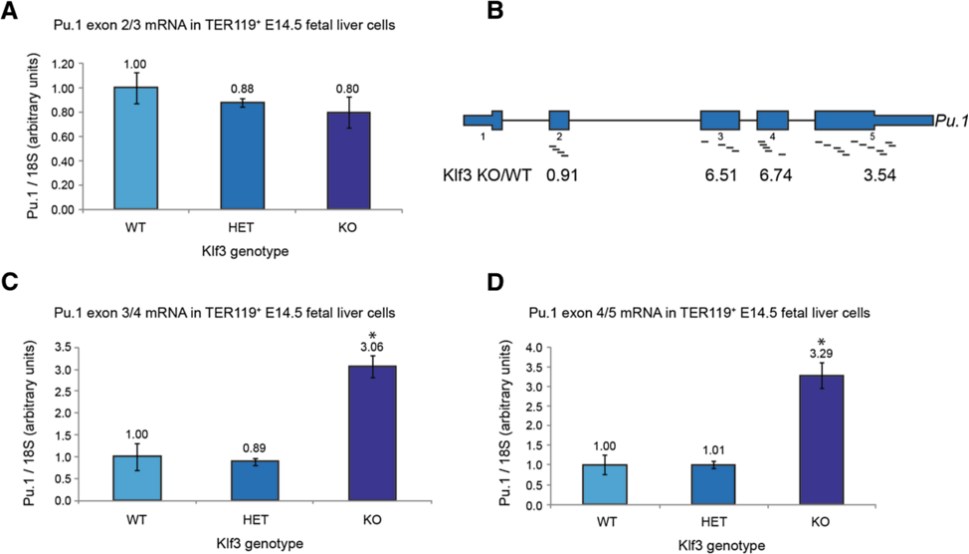
**Transcripts containing exons towards the 3′ end of *Pu.1 *are upregulated in *Klf3***^**−/− **^**E14.5 TER119**^**+ **^**fetal liver cells. (A, C, D)** Transcript levels in *Klf3*^+/+^ (WT, *n* = 2), *Klf3*^*+/−*^ (HET, *n* = 3), and *Klf3*^−/−^ (KO, *n* = 3) cells were determined by quantitative real-time RT-PCR using forward and reverse primer combinations specific for exons 2 and 3 **(A)**, exons 3 and 4 **(C)**, or exons 4 and 5 **(D)** of *Pu.1*. Values have been normalized to *18S* rRNA levels and in each instance the *Klf3*^+/+^ sample has been set to 1.0. Error bars represent standard error of the mean. *, *P* < 0.02 compared to both *Klf3*^+/+^ and *Klf3*^*+/−*^ (Student’s two-tailed *t*-test). **(B)** Positions of microarray probes across the *Pu.1* gene and their relative intensities in *Klf3*^−/−^ compared to *Klf3*^+/+^ samples. Exons are displayed as blue boxes and are widened to denote the coding region. Schematic is not to scale.

We first sought to validate the upregulation of *Pu.1* expression in *Klf3*^−/−^ cells by quantitative real-time RT-PCR. Initial experiments, using primers that span the exon 2/3 junction of *Pu.1*, did not recapitulate the microarray results (Figure 1A). Unexpectedly, *Pu.1* mRNA was detected at similar levels in *Klf3*^+/+^, *Klf3*^+/−^, and *Klf3*^−/−^ Ter119^+^ E14.5 fetal liver cells. To resolve this discrepancy, we analyzed the individual probe intensities across the *Pu.1* locus from the microarray data. The murine *Pu.1* gene comprises five exons and of these, exons 2 to 5 are represented by probes on the arrays. Expression of only exons 3 to 5 of *Pu.1* was found to be higher in *Klf3*^−/−^ compared to *Klf3*^+/+^ tissue; however, expression of exon 2 was unchanged (Figure 1B). Real-time RT-PCR using primers specific for the exon 3/4 and exon 4/5 boundaries of *Pu.1* (Figure 1C, D) confirmed that indeed, exons 3 to 5 exhibit upregulated expression in *Klf3*^−/−^ cells while exon 2 does not (Figure 1A).

### An *ORR1A0* LTR element serves as an alternative promoter in the *Pu.1* locus in the absence of KLF3

**Figure 2:**
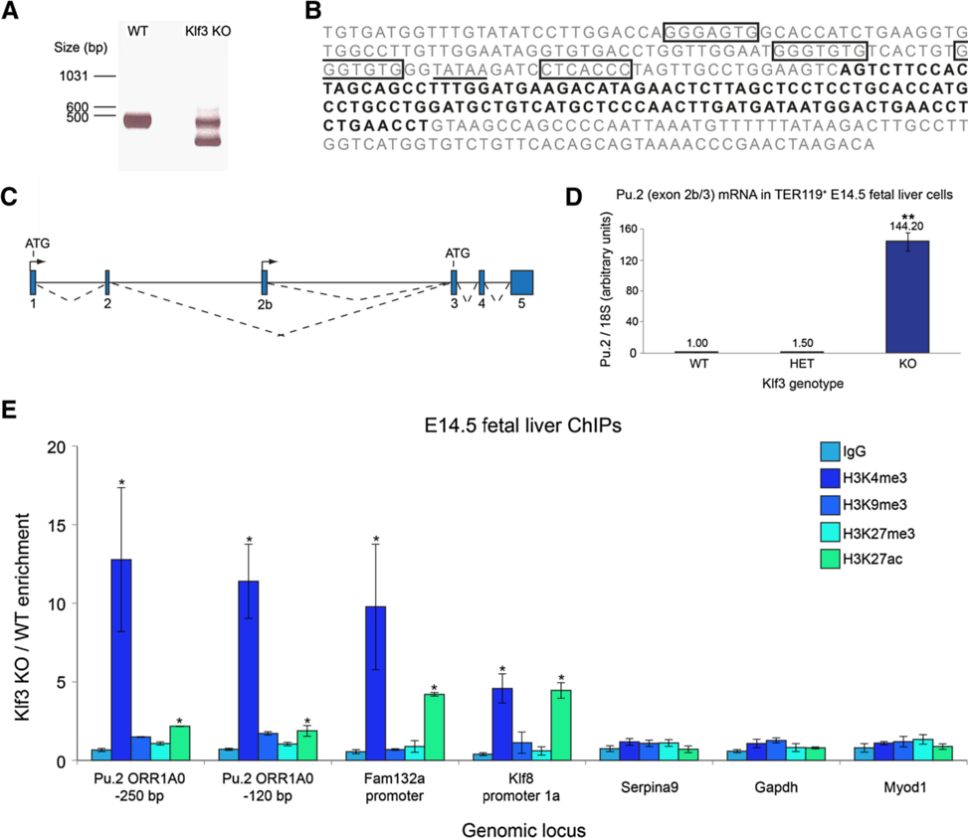
**A novel, internal *Pu.1 *promoter resides within an *ORR1A0 *LTR element and is repressed by KLF3. (A)** RNA from *Klf3*^+/+^ (WT) and *Klf3*^−/−^ (KO) TER119^+^ fetal liver cells was subjected to 5′ RACE using a reverse primer specific for exon 3 of *Pu.1* and analyzed by agarose gel electrophoresis. The smaller band in the *Klf3*KO lane was sequenced and found to contain a novel exon (exon 2b). **(B)** The sequence of the *ORR1A0* LTR, in which *Pu.1* exon 2b is shown in bold. Sequences which fit the KLF binding consensus 5′-NCN CNC CCN-3′ are boxed, and the TATA box at −30 is underlined. **(C)** Schematic of the murine *Pu.1* locus showing the position of exon 2b. Exons are represented by blue boxes, transcription start sites by arrowheads and splicing events by broken lines. Start points of translation (ATGs) for the two alternative transcripts are also shown. **(D)** Real-time RT-PCR quantification revealing that transcripts containing exon 2b spliced to exon 3 of *Pu.1* (that is, *Pu.2* transcripts) are upregulated in *Klf3*^−/−^ TER119^+^ E14.5 fetal liver cells compared to *Klf3*^+/−^ (HET) and *Klf3*^+/+^. Values have been normalized to *18S* rRNA and the *Klf3*^+/+^ sample has been set to 1.0. *n* = 3 for each genotype. **, *P* <0.005 compared to both *Klf3*^+/+^ and *Klf3*^*+/−*^ (Student’s two-tailed *t*-test). **(E)** ChIPs were performed on *Klf3*^+/+^ and *Klf3*^−/−^ E14.5 fetal livers (*n* = 2 or 3 of each genotype per IP). Data are represented as the fold-change enrichment in *Klf3*^−/−^ cells compared to *Klf3*^+/+^. The *Fam132a* and *Klf8* promoters have been included as positive controls while *Serpina9*, *Gapdh*, and *MyoD* are negative control regions. *, *P* <0.05 compared to *Gapdh* (Student’s one-tailed *t*-test). In **(D and E)**, error bars represent standard error of the mean.

The upregulated expression of exons towards the 3′ end of *Pu.1* raised the possibility that an alternative, internal promoter was driving transcription from the locus and that this promoter is repressed by KLF3. To investigate this, we conducted 5′ RACE on mRNA from *Klf3*^+/+^ and *Klf3*^−/−^ Ter119^+^ fetal liver cells using a reverse primer specific for exon 3 of *Pu.1*. While electrophoretic separation of RACE products revealed a common transcript in both samples (an approximately 420 bp band), a smaller transcript (226 bp) was found in the *Klf3*^−/−^ sample (Figure 2A). Sequencing of the two RACE products revealed that the larger band corresponds to exons 1 to 3 of a typical *Pu.1* transcript (GenBank:NM_011355). The shorter transcript, however, was found to contain exon 3 of *Pu.1* preceded by a novel sequence (shown in bold in Figure 2B). This sequence maps to intron 2 of *Pu.1* and represents an alternative leader exon, hereby termed exon 2b, which is spliced to exon 3 and which has not been documented previously, to our knowledge (Figure 2C). Hereafter, we refer to this novel transcript as *Pu.2*.

Searches using the RepeatMasker program showed that exon 2b lies within a 343 bp long terminal repeat (LTR) element, named *ORR1A0*, belonging to the *MaLR* (*mammalian apparent LTR retrotransposon*) family (Figure 2B) [52]. The *ORR1A0* element in the murine *Pu.1* locus contains several hallmarks of a eukaryotic core promoter including a TATA box at −30, an initiator sequence (5′-TCAGTY-3′) at the TSS and a downstream promoter element around +30 [53]. In addition, it contains several motifs fitting the KLF DNA-binding consensus 5′-NCN CNC CCN-3′ (Figure 2B).

In order to verify that this novel *Pu.2* transcript is de-repressed in erythroid cells lacking KLF3, we performed real-time RT-PCR on *Klf3*^+/+^, *Klf3*^+/−^ and *Klf3*^−/−^ Ter119^+^ E14.5 fetal liver RNA using a forward primer specific for exon 2b and a reverse primer targeting exon 3 of *Pu.1*. Indeed, significant up-regulation of the *Pu.2* transcript (>140-fold) was observed in *Klf3*^−/−^ compared to *Klf3*^+/+^ and *Klf3*^+/−^ samples (Figure 2D). While this transcript was not amplified from wild-type tissue by 5′ RACE (Figure 2A), we detected low amounts of it in wild-type and *Klf3*^+/−^ tissue by RT-PCR. In adult *Klf3*^−/−^ mice, we observed marked upregulation of this chimeric transcript in erythroid organs (spleen and bone marrow) (Additional file 1: Figure S1A). In contrast, canonical *Pu.1* mRNA was unaltered in these and other tissues examined (Additional file 1: Figure S1B).

KLF3 can repress transcription by recruiting CTBP, a co-repressor that silences genes through a number of different histone-modifying enzymes. We therefore analyzed a series of histone marks around the *Pu.2 ORR1A0* promoter in *Klf3*^−/−^ compared to *Klf3*^+/+^ E14.5 fetal liver cells (Figure 2E). In particular, we observed a marked increase specifically of histone 3 lysine 4 tri-methylation (H3K4me3) in *Klf3*^−/−^ cells at the *ORR1A0* promoter (approximately 12-fold). We found that this mark was also increased at the promoters of previously validated KLF3 target genes such as *Klf8*[50] and *Fam132a*/*adipolin*[49,54] (Figure 2E). H3K4me3 is a mark typically found at actively transcribed promoters [55]. Moreover, loss of this mark, rather than the acquisition of repressive modifications, has been reported during the developmental silencing of retroelements [18]. In addition, the *Pu.2* promoter displayed only a moderate level of H3K9me3 in wild-type cells (Additional file 2: Figure S2). This was not appreciably altered in *Klf3*^−/−^ cells, suggesting that H3K9 tri-methylation is not the primary mechanism through which KLF3 silences transcription at this locus. Together, these results indicate that the *ORR1A0* element is a functional, alternative promoter for the *Pu.1* gene in erythroid cells and is highly de-repressed in the absence of KLF3.

### KLF1 and KLF3 can bind to the CACCC-like boxes in the *ORR1A0* LTR and activate and repress transcription, respectively

**Figure 3:**
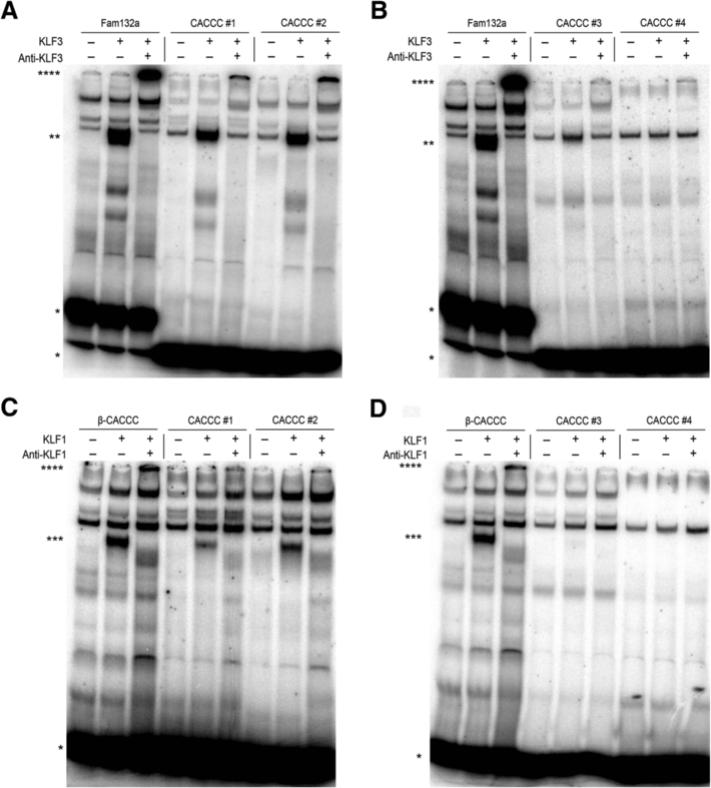
**KLF1 and KLF3 bind to CACCC boxes in the *ORR1A0 *LTR.** Nuclear extracts were harvested from COS cells expressing KLF3 **(A, B)** and KLF1 **(C, D)** and were analyzed by EMSA using radiolabelled probes covering the four CACCC boxes in the *ORR1A0* LTR promoter shown in Figure 2b. Unbound DNA probes are indicated by *. KLF3:DNA and KLF1:DNA complexes are represented by ** and ***, respectively. The identities of these complexes were confirmed by supershifting (****) with antibodies specific for KLF3 **(A, B)** and KLF1 **(C, D)**. In **(A and B)**, a radiolabelled probe encompassing a known KLF3 binding site in the *Fam132a* promoter [54] has been included as a positive control. In **(C and D)**, a probe containing a CACCC-box from the *β-major globin* promoter, a site that is strongly bound by KLF1 [46], has been used as a positive control. In **(A and B)**, a background band present in mock-transfected COS cells co-migrates with KLF3, but at a much lower intensity and it does not shift with the KLF3 antibody.

We next examined by electrophoretic mobility shift assay (EMSA) whether one or more of the four 5′-NCN CNC CCN-3′ sites in the *ORR1A0* promoter are recognized by KLF3. Indeed, KLF3 was found to bind strongly to the two sites most distal to the TSS and weakly to the third CACCC-box (Figure 3A, B). We next assessed whether the related family member KLF1 also binds to these sites. KLF1 is highly expressed in erythroid cells and has a similar DNA-binding specificity to KLF3, such that the two proteins co-regulate overlapping genes *in vivo*[46,49]. We found that like KLF3, KLF1 binds to the two 5′ most sites (Figure 3C). No detectable binding was observed for the CACCC boxes closest to the TSS (Figure 3D).

**Figure 4:**
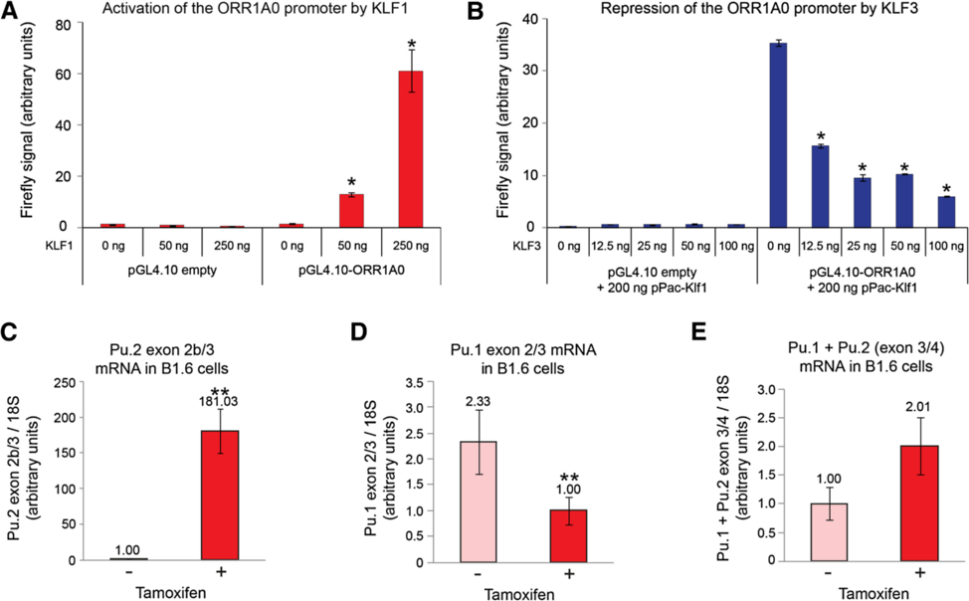
**KLF1 drives expression from the *Pu.2 ORR1A0 *promoter. (A, B)** SL2 cells were co-transfected with pGL4.10 *Firefly* luciferase reporter (promoter-less or containing the *ORR1A0* promoter) together with increasing amounts of pPac-Klf1 **(A)** or a steady amount of pPac-Klf1 and increasing dosage of pPac-Klf3 **(B)**. *Firefly* levels have been normalized to *Renilla* luciferase and in each instance the lowest value has been set to 1.0. Charts represent the mean of triplicate experiments and error bars show standard error of the mean. *, *P* <0.005 (Student’s two-tailed *t*-test) compared to pGL4.10-*ORR1A0* wells transfected with 0 ng pPac-Klf1 **(A)** or 0 ng pPac-Klf3 **(B)**. **(C**-**E)** KLF1-ER activity was induced in B1.6 cells by addition of tamoxifen and total RNA was extracted after 48 h and analyzed by quantitative real-time RT-PCR. Transcripts containing exons 2b/3 are increased **(C)** while those containing exons 2/3 are decreased **(D)**. The total pool of *Pu.1* plus *Pu.2* mRNA is represented by exons 3/4 **(E)**. Values have been normalized to *18S* rRNA and in each case, the lowest value has been set to 1.0. Error bars represent standard error of the mean and *n* = 4 for each condition. **, *P* <0.05 (Student’s paired two-tailed *t*-test) compared to untreated cells.

Having established that both KLF1 and KLF3 can bind to motifs present in the *ORR1A0* element, we next assessed whether they can functionally regulate this promoter in cellular assays. To do this, we cloned the *ORR1A0* promoter upstream of a *Firefly* luciferase reporter gene in the pGL4.10[*luc2*] vector. This was then co-transfected together with increasing amounts of KLF1 in SL-2 cells, a cell line that is often used to examine KLF function due to minimal background CACCC-binding activity [45]. We found that KLF1 strongly activates expression from the *ORR1A0* promoter but has little effect on empty pGL4.10[*luc2*] vector (Figure 4A). By titrating increasing dosage of KLF3 we found that it counters the activity of KLF1 at the *ORR1A0* promoter and represses expression (Figure 4B).

To investigate whether KLF1 indeed drives transcription of these chimeric *Pu.2* transcripts from the *ORR1A0* element *in vivo*, we employed a KLF1-inducible erythroid cell line known as B1.6 [56]. These cells were derived from *Klf1*^−/−^ fetal liver and have been rescued with a transgene encoding a tamoxifen-inducible KLF1-ER (estrogen receptor) fusion protein. Upon addition of tamoxifen, KLF1-ER is activated and drives expression of KLF1 target genes to induce hemoglobinization and erythroid differentiation. KLF3 protein has not been detected in these cells, although *Klf3* mRNA is induced after KLF1-ER activation [50].

Using real-time RT-PCR we observed a low level of *Pu.2* mRNA in untreated B1.6 cells; however, tamoxifen induction resulted in a dramatic increase of these transcripts (Figure 4C). Moreover, despite decreased expression of canonical *Pu.1* transcripts upon KLF1-ER induction (represented by exon 2/exon 3, Figure 4D), the total level of *Pu.1* plus *Pu.2* transcripts increased (represented by the exon 3/exon 4 junction, Figure 4E), albeit not significantly. This suggests that the chimeric transcripts contribute substantially to the total *Pu.1* plus *Pu.2* mRNA pool in induced B1.6 cells. In addition, the induction of *Pu.2* expression was rapid following tamoxifen addition (within 2 hours) and occurred in the presence of the translation inhibitor cycloheximide (Additional file 3: Figure S3A, B). Taken together, these data suggest that KLF1 directly activates *Pu.2* transcription from the endogenous *ORR1A0* promoter in erythroid cells in the absence of KLF3.

### Widespread de-repression of chimeric transcripts from *ORR1A0* elements in the absence of KLF3

A RepeatMasker survey revealed that there are approximately 2,130 *ORR1A0* integrants in the mouse genome. The consensus sequence of *ORR1A0* found in Repbase contains all of the core promoter sequences shown in Figure 2B as well as the four 5′-NCN CNC CCN-3′ motifs. In addition, there is little divergence between individual *ORR1A0* elements with elements generally sharing greater than 97% sequence identity to the consensus [57]. We therefore hypothesized that KLF3 might play a broader role in silencing aberrant transcription from *ORR1A0* LTRs.

To investigate this, we performed RNA-Seq on triplicate samples of *Klf3*^*+/+*^ and *Klf3*^−/−^ E14.5 TER119^+^ fetal liver cells. In total, 1,025 genes were found to be significantly deregulated (FDR <0.05) in the absence of KLF3 (Additional file 4: Table S1). The majority of these (76.7%) were upregulated in *Klf3*^−/−^ cells, concordant with the view from previous studies that KLF3 is predominantly a transcriptional repressor [49]. Importantly, previously validated KLF3 targets also displayed significant upregulation by RNA-Seq including *Klf8* (108-fold), *Lgals3* (33-fold), *Fam132a*/*adipolin* (7.5-fold), *Hba-x* (2.3-fold), and *Hbb-y* (1.8-fold) [49,50,54,58].

We next assessed whether the *ORR1A0* LTR, and related *ORR1A0-int*, elements were enriched among the list of KLF3 target genes. We found that of the 786 significantly upregulated genes, 166 of these (21.1%) contained one or more *ORR1A0* LTRs. In contrast, of the 239 downregulated genes, there was only one instance of an overlap with an *ORR1A0* element (0.004%). Similarly, *ORR1A0-int* elements, which are typically flanked by *ORR1A0* LTRs, were found in 96 upregulated genes (12.2%) and only in a single case of a downregulated gene. These results illustrate a clear enrichment of the *ORR1A0* and *ORR1A0-int* retroelements specifically within genes that are normally repressed by KLF3 in erythroid cells.

Because of sequence conservation between *ORR1A0* elements and difficulties associated with unambiguously assigning them to specific genomic loci, we instead looked for evidence of splicing between *ORR1A0* transcripts and downstream genic exons. To do this, we confined our analysis to annotated genes which displayed differential isoform expression in *Klf3*^−/−^ cells (greater than 10-fold upregulated compared to *Klf3*^+/+^). We identified 70 such genes (Additional file 5: Table S2). Of these, 34.3% contained transcribed *ORR1A0* elements, and almost half of these (41.7%) were spliced to genic exons and a further 16.7% showed splicing to un-annotated exons.

**Figure 5:**
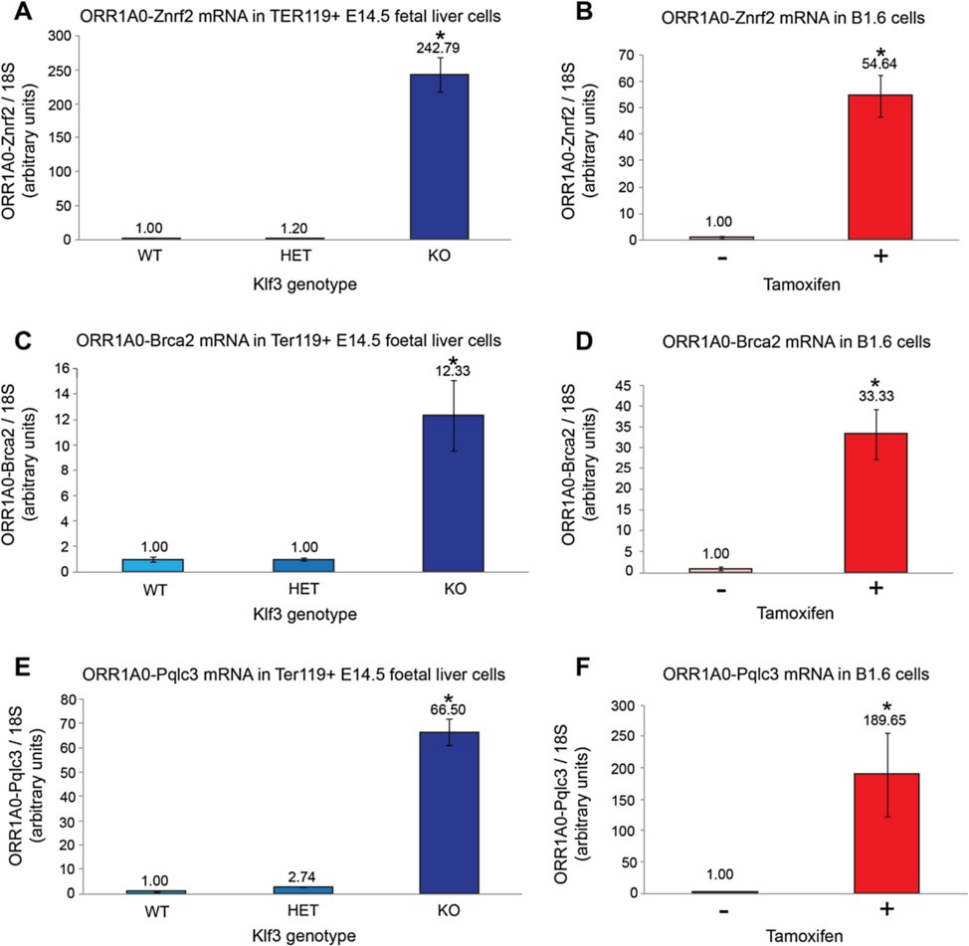
**KLF1 activates while KLF3 represses chimeric transcripts from *ORR1A0 *LTRs in erythroid cells.** RNA from *Klf3*^+/+^ (WT), *Klf3*^+/−^ (HET), and *Klf3*^−/−^ (KO) TER119^+^ E14.5 fetal liver cells **(A, C, E)** and from untreated and tamoxifen-treated KLF1-ER inducible B1.6 cells **(B, D, F)** was analyzed by quantitative real-time RT-PCR using forward primers which recognize the *ORR1A0* exon and reverse primers specific for downstream exons of the *Znrf2***(A, B)**, *Brca2***(C, D)** and *Pqlc3***(E, F)** genes. All values have been normalized to *18S* rRNA levels and WT **(A, C, E)** and untreated **(B, D, F)** samples have been set to 1.0. Error bars represent standard error of the mean and *n* = 3 for each genotype or condition. *, *P <*0.05 (Student’s two-tailed *t*-test) compared to both *Klf3*^+/+^ and *Klf3*^+/−^**(A, C, E)** and compared to untreated cells **(B, D, F)**.

By real-time RT-PCR, we validated these results for a selection of candidate target genes. Using forward primers specific for the *ORR1A0* exon and reverse primers specific for downstream genic exons, we observed striking upregulation for all three genes tested (*Znrf2*, *Brca2*, and *Pqlc3*) in E14.5 TER119^+^ cells lacking KLF3 (Figure 5A, C, E), mirroring our previous result for *Pu.2* transcripts (Figure 2D). In addition, expression of all of these chimeric mRNAs increased considerably upon tamoxifen induction of B1.6 cells (Figure 5B, D, F). In these cells, their upregulation was rapid (Additional file 3: Figure S3C, E) and occurred in the presence of cycloheximide (Additional file 3: Figure S3D, F), suggesting that like *Pu.2*, their transcription is also directly driven by KLF1. Lastly, it should be noted that definitively mapping repetitive reads to their correct genomic loci is difficult and thus the RNA-Seq analysis is not anticipated to give an exhaustive list of genes for which *ORR1A0* exons are spliced to downstream exons. Indeed, by targeted real-time RT-PCR we assessed a further five candidate genes for which the RNA-Seq analysis had not called splicing events (*Cd59b*, *Tmx4*, *Bzw2*, *Cpe*, and *Tcfl5*). In each case, we found that in *Klf3*^−/−^ cells, the *ORR1A0* exon is spliced and the resulting chimeric transcripts are markedly upregulated compared to *Klf3*^+/+^ and *Klf3*^+/−^ cells (Additional file 6: Figure S4A-C).

**Figure 6:**
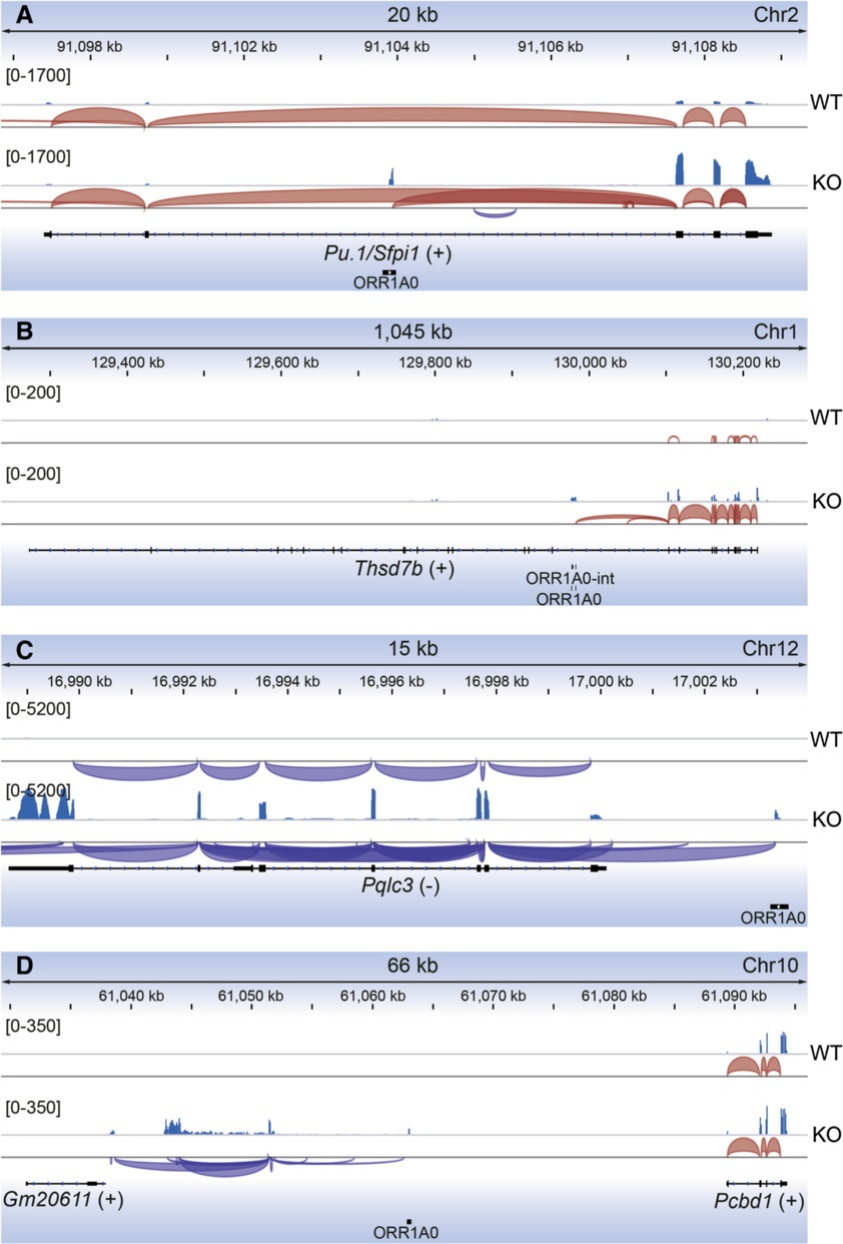
**RNA-Seq analysis of de-repressed chimeric transcripts in erythroid cells in the absence of KLF3.** Tracks represent merged data for triplicate *Klf3*^+/+^ (WT) and *Klf3*^−/−^ (KO) E14.5 TER119^+^ fetal liver cell samples. Four loci are shown: the *Pu.1/Sfpi1* gene **(A)**, the *Thsd7b* gene **(B)**, the *Pqlc3* gene **(C)**, and a region on chromosome 10 **(D)**. In each panel, sequencing reads are shown for WT (top) and KO (bottom). Within each panel, the intensity scale is consistent for both genotypes and is shown on the left. Underneath the reads, detected splicing events are shown in red (sense) or purple (anti-sense). Similarly, directionality of genes has been denoted as being on either the sense (+) or anti-sense (−) strand. The positions of *ORR1A0* and *ORR1A0-int* elements are shown at the bottom of each panel. In **(A and B)**, internal *ORR1A0*s are transcribed and spliced to downstream genic exons which show a marked increase in expression in KO samples. In **(C)**, an *ORR1A0* element serves as an upstream promoter and transcripts are spliced to genic exons. In **(D)**, an *ORR1A0* is transcribed solely in KO cells and is spliced to unannotated exons.

De-repressed transcription from *ORR1A0* elements was found to affect local gene expression in a number of ways, shown in Figure 6 and Additional file 7: Figure S5. In many instances, *ORR1A0* LTRs reside within the body of the gene and the new transcripts are spliced to downstream genic exons. This is the case for *Pu.1*, *Thsd7b*, *Znrf2*, and *Brca2* (Figure 6A, B, Additional file 7: Figure S5A, B). In addition, *ORR1A0* LTRs upstream of genes also act as novel transcriptional start sites for such chimeric transcripts, as is the case for *Pqlc3* (Figure 6C). We also observed spliced transcripts emanating from *ORR1A0* elements in un-annotated regions (Figure 6D) and also detected novel transcripts antisense to known genes (Additional file 7: Figure S5C). Lastly, in several cases we observed significantly de-repressed transcription from and across *ORR1A0* elements that did not appear to influence the expression of the surrounding gene, as for *Drosha* (Additional file 7: Figure S5D). This typically occurred either where two *ORR1A0* LTRs exist as a cassette, flanking an *ORR1A0-int* element (Additional file 7: Figure S5D), or where they are in an antisense direction to a transcribed gene (Additional file 7: Figure S5C).

Several of these chimeric transcripts have previously been reported as ESTs that have typically been detected in embryonic cells and tissues from a range of developmental stages including 4-cell (*Brca2*; GenBank:CN716605) and 8-cell stage embryos (Chr10: chr10:61042355–61063209 shown in Figure 6D; GenBank:CJ067427), E13 liver tissue (chr9:9049867–9081010; GenBank:CJ043932), and E17 amnion (*Znrf2*; GenBank: BY073363 and CJ093793). This provides further evidence that *ORR1A0* LTRs are capable of functioning as *bona fide* promoters *in vivo*.

### The chimeric *Pu.2* transcript driven by the *ORR1A0* promoter is translated *in vivo*

Thus far, the results presented here suggest that KLF3 silences aberrant transcription from *ORR1A0* LTRs in erythroid cells. These chimeric transcripts potentially encode protein variants that might functionally impact normal murine erythropoiesis. Indeed, *Klf3* null mice exhibit a number of erythroid defects including increased immature red blood cells (reticulocytes) and nuclear inclusions (Howell-Jolly bodies) in peripheral blood [49]. We thus sought to determine whether these chimeric transcripts are in fact translated *in vivo* using the *Pu.1* gene as an example, given the role of this transcription factor as a master regulator of hematopoietic differentiation [59].

**Figure 7:**
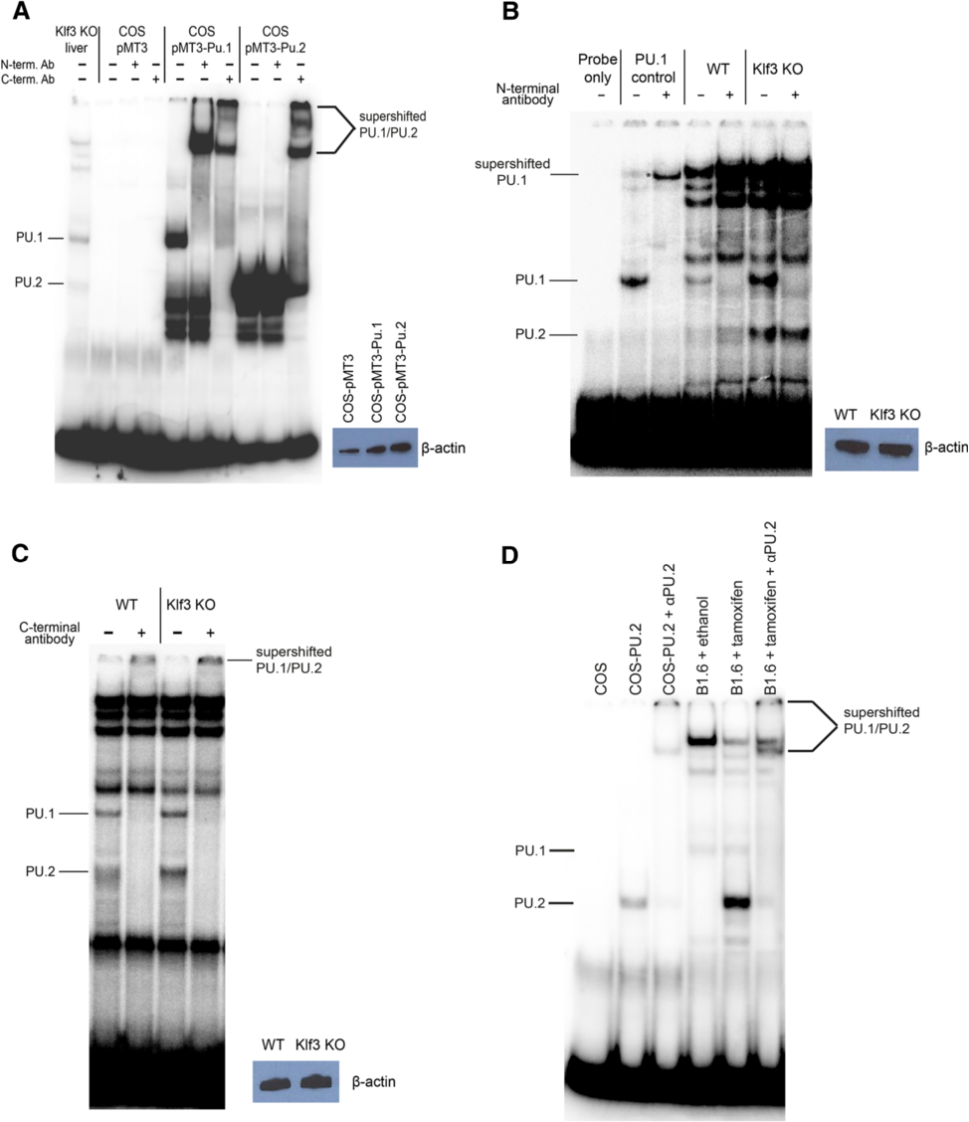
**PU.2 is a LTR-driven novel isoform of PU.1 that retains DNA-binding activity.** Nuclear extracts were analyzed by EMSA using a radiolabelled probe containing the PU.1 DNA-binding consensus. **(A)** PU.1 and PU.2 expressed in COS cells co-migrate with bands observed in *Klf3*^*−/−*^ (KO) fetal liver nuclear extracts. PU.1 is supershifted by antibodies specific for the N-terminus and C-terminus, while PU.2 is only recognized by the C-terminal antibody. Nuclear extracts from COS cells transfected with empty pMT3 vector have been included as a control. **(B, C)** In *Klf3*^+/+^ (WT) and *Klf3*^−/−^ (KO) fetal liver nuclear extracts, the band which co-migrates with PU.1 is recognized by both antibodies while the band that co-migrates with PU.2 is only supershifted by the C-terminal antibody, confirming the identities of the two bands. In **(A-C)**, comparative quantification of nuclear extract preparations was achieved by western blotting for β-actin. **(D)** Nuclear extracts from untreated and tamoxifen-treated KLF1-ER inducible B1.6 cells. Nuclear extracts from COS cells transfected with PU.2 (and mock transfected) have been included as controls. The identity of the PU.2 is confirmed by addition of the C-terminal antibody (αPU.2). In **(A**-**D)**, supershifts have been indicated by arrows, and additionally by an asterisk in **(B)**.

The chimeric *Pu.2* transcript contains a potential ATG start codon within exon 3 (Figure 2C) and is predicted to encode a truncated isoform (PU.2) that lacks 88 amino acids at its N-terminus. Since the ETS DNA-binding domain lies at the C-terminus of PU.1, we anticipated that PU.2 would retain DNA-binding ability. We cloned and expressed PU.1 and PU.2 in COS cells and tested their ability to bind to a radiolabelled probe containing the PU.1 DNA-binding consensus sequence (5′-GAGGAA-3′) by EMSA. Indeed, PU.2 is able to bind to DNA and migrates more rapidly than PU.1 (Figure 7A). Moreover, while PU.1 is recognized and supershifted by antibodies raised against both the N- and C-terminus of PU.1, PU.2 is only supershifted by the antibody specific for the C-terminus (Figure 7A).

To determine whether PU.2 protein is expressed *in vivo*, we analyzed nuclear extracts from E14.5 *Klf3*^−/−^ fetal livers. Extracts from these cells formed bands that co-migrated with both PU.1 and PU.2 (Figure 7A). The upper band is supershifted by both antisera while the lower band is only supershifted by the C-terminal antisera, confirming their identities as PU.1 and PU.2, respectively (Figure 7B, C). PU.2 protein was also detected in nuclear extracts from *Klf3*^+/+^ fetal liver cells (Figure 7B, C) albeit at a lower level than in *Klf3*^−/−^ samples. Lastly, we also observed marked induction of PU.2 protein upon tamoxifen activation of KLF1-ER in B1.6 cells (Figure 7D) consistent with the upregulation of *Pu.2* transcripts (Figure 4C). Taken together, these results indicate that PU.2 is indeed translated in erythroid cells *in vivo*.

### PU.2 can act as a dominant negative protein in erythroid cells

The PU.2 protein lacks the N-terminal activation domain of PU.1, a region that interacts with the general transcription factor TFIID [60]. We therefore postulated that PU.2 might not function as a transcriptional activator and might antagonize the activity of PU.1 at its target genes. To investigate this, we first conducted reporter assays using the promoter of a previously characterized PU.1 target gene, *CLEC5A*[61]. We found that while PU.1 robustly activated expression, PU.2 repressed this promoter in a dose-dependent manner (Additional file 8: Figure S6A, B).

**Figure 8:**
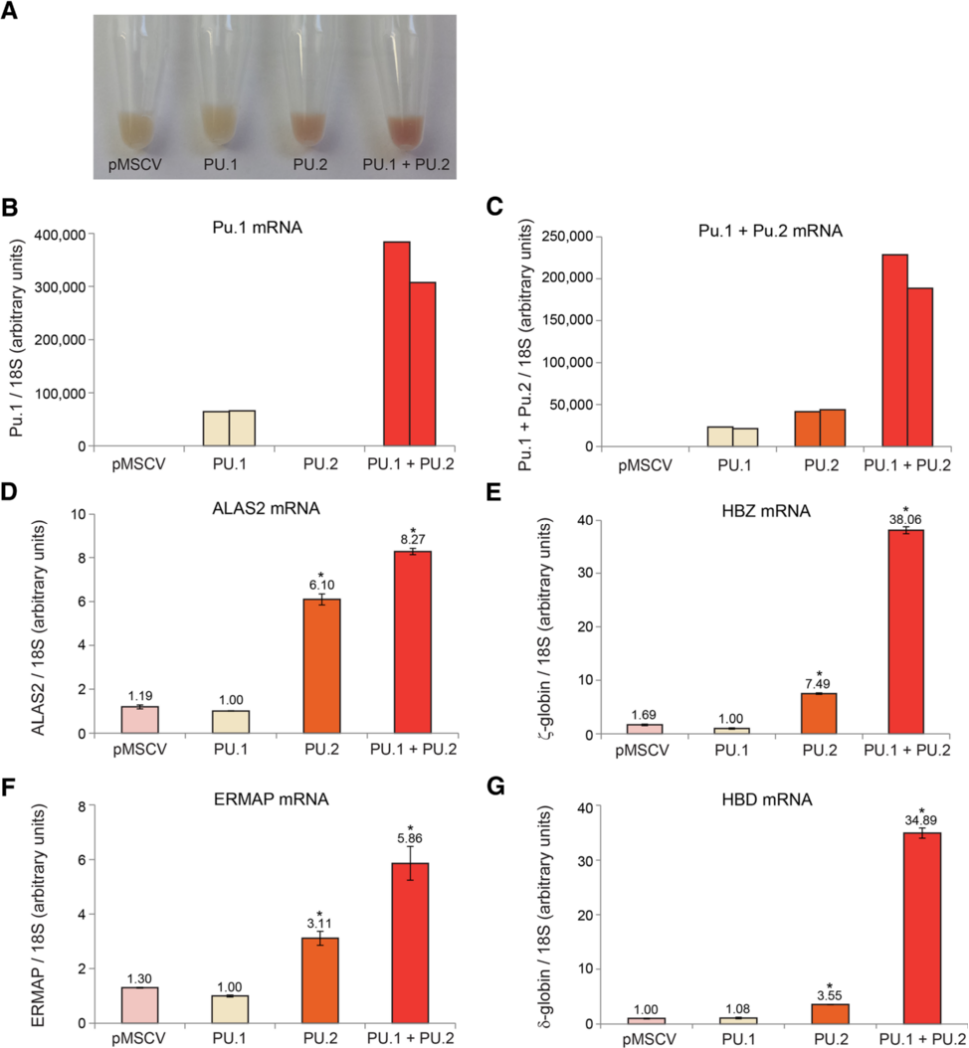
**Forced expression of PU.2 in K562 cells induces spontaneous erythroid differentiation. (A)** Cell pellets of K562 lines infected with pMSCVpuro-Pu.1, pMSCVhyg-Pu.2, pMSCVpuro-Pu.1 plus pMSCVhyg-Pu.2, and empty vector (pMSCVpuro plus pMSCVhyg). Hemoglobinization (signified by red hue) is apparent in the lines expressing PU.2 and PU.1 plus PU.2. **(B-G)** Total RNA was extracted from duplicate lines and was subjected to quantitative real-time RT-PCR analysis using primers specific for: **(B)** the 5′ end (exon 2/3) of the murine *Pu.1* gene (recognizes *Pu.1* only and not *Pu.2*); **(C)** the 3′ end (exon 3/4) of *Pu.1*/*Pu.2* (recognizes both *Pu.1* and *Pu.2*); **(D)***ALAS2*; **(E)***ζ-globin* (*HBZ*); **(F)***ERMAP*; and **(G)***δ-globin* (*HBD*). In **(B and C)** the duplicate lines have been shown separately while in **(D-G)** the average of the two lines has been determined. Levels have been normalized to *18S* rRNA and set to 1.0 for the lowest samples. Error bars represent standard error of the mean. *, *P* <0.05 (Student’s two-tailed *t*-test) compared to empty vector.

We next sought to examine the possible dominant negative activity of PU.2 in a hematopoietic system. To do this, we ectopically expressed PU.2, with or without PU.1, in human K562 cells and derived stable clones (Figure 8B, C). Forced expression of PU.1 has previously been shown to promote monocytic differentiation of these cells while inhibiting erythroid maturation [62]. Strikingly, we found that expression of PU.2, both by itself and when co-expressed with PU.1, caused spontaneous erythroid differentiation of these cells in the absence of any chemical-inducing agents (Figure 8A). Microarray analysis and real-time RT-PCR validation of these cell lines confirmed the upregulation of multiple erythroid genes including the *globins*, *ALAS2*, and *erythroblast membrane-associated protein* (*ERMAP*) (Additional file 9: Table S3, Figure 8D-G). These results suggest that the LTR-driven PU.2 protein that is upregulated in the absence of KLF3 can oppose the normal function of PU.1 and promote erythroid differentiation.

## Discussion

It has recently been shown that TEs frequently act as promoters of genic transcription and are dynamically transcribed during ontogeny [17,18,20,22]. Typically, the majority of retroelement silencing occurs early in gestation [28] and numerous studies have demonstrated the role of histone- and DNA-modifying enzymes in this process [27,30-34,36]. KRAB domain zinc finger proteins, which interact with the co-repressor TRIM28, have been proposed to play a role in the specific recognition and repression of distinct retroelement families [31,35,37,39,40]. This has indeed shown to be the case for ZFP809 in embryonic stem cells [38]. Other DNA-binding zinc finger proteins, including REX1/ZFP42, have also been implicated, but their mechanism of action remains unclear and they appear to affect multiple, unrelated retroviral families [63].

Here we show that the zinc finger protein KLF3, which lacks a KRAB domain, is required for the silencing of transcription from *ORR1A0* elements of the *MaLR* family. In the absence of KLF3, there is a pronounced increase in chimeric transcripts generated from these LTRs. The phenomenon of deregulated chimeric transcription has previously been observed upon ablation of epigenetic modifiers such as SETDB1 and LSD1 in embryonic stem cells [27,34]. For example, in cells lacking SETDB1, 15% of de-repressed genes arise due to failed silencing of promoter-proximal ERVs and half of these genes exhibit chimeric transcripts [27]. However, in both of these cases the effects described were more global than we observe for KLF3 and covered diverse retroelement families.

Silencing of the *ORR1A0* LTR by KLF3 appears to occur largely independently of the TRIM28/SETDB1 pathway and DNA methylation. Analysis of over 23,000 TRIM28 binding sites in ES cells compiled from two studies [40,64] revealed that only eight lie within 100 bp of an *ORR1A0* element. This may partially reflect the difficulties of detecting ChIP peaks that extend beyond the boundaries of repeat elements. However, a separate examination of regions of TRIM28-dependent H3K9 tri-methylation, which typically spread beyond repeat borders, revealed that only 62 of the 2,140 *ORR1A0* LTRs (that is, 2.9%) lie within 100 bp [40]. Similarly, analysis of the DNA methylation status of hematopoietic stem cells and erythroblasts revealed that only 1.0% (22) and 0.3% (7) of *ORR1A0*s, respectively, lie proximal to regions of DNA-methylation as determined by MBD-Seq [65].

From a number of *Klf3*^−/−^ tissues examined, the upregulation of *ORR1A0* transcription appeared to be restricted to erythroid cells. This is of particular interest given that most retroelement silencing has been demonstrated in embryonic stem and germ cells [27,30,31,33]. This suggests that KLF3 plays an active role in somatic repression of retroviral transcription, both in fetal and adult erythroid tissues. Consistent with its erythroid restricted profile, we found that the master erythroid regulator KLF1 drives expression of *ORR1A0*-originating transcripts in the absence of KLF3.

The specific recognition of the *ORR1A0* element by KLF3 and KLF1 appears to involve two 5′-CACNCCC-3′ boxes upstream of the TSS (Figures 2B and 3). The similar DNA-binding specificities of KLF1 and KLF3 have previously been noted and indeed, approximately 50% of KLF3 target genes in erythroid cells are also regulated by KLF1 [49]. Interestingly, the *ORR1A0*-related LTR *ORR1A1* lacks one of the 5′-CACNCCC-3′ boxes and additionally lacks the TATA box. Despite being 97% homologous with *ORR1A0* and occurring at twice the frequency in the mouse genome, we did not observe enrichment of *ORR1A1* in KLF3 repressed transcripts, alluding to the functional importance of these two promoter motifs and the extraordinary specificity of KLF3 for the *ORR1A0* LTR.

Moreover, although the *ORR1* retroelement family is abundantly represented throughout diverse rodent species, the *ORR1A0* LTR is specifically only found in the mouse. It is interesting to note that the DNA-binding domain of KLF3 shows complete sequence conservation between *Mus musculus*, *Rattus norvegicus*, and *Homo sapiens*. This suggests that in the mouse, the DNA-binding specificity of KLF3 has not altered in response to the emergence of the *ORR1A0* retroelement. Rather, it appears that KLF3-mediated repression of *ORR1A0* occurred intrinsically from the initial appearance of the retroelement, and in this context, the spread of the *ORR1A0* LTR may have been tolerated without deleterious impacts.

While KLF3 appears to efficiently silence *ORR1A0* transcription in spleen and bone marrow cells, it is possible that at particular stages of development or cellular maturation, KLF1 predominates and these chimeric transcripts are expressed at higher levels, as observed in the B1.6 erythroblast line. These chimeric transcripts potentially encode functional protein isoforms and indeed, in the case of the *Pu.1* locus, we have detected a truncated isoform expressed in fetal liver. PU.2 counters the normal activity of PU.1 and instead promotes erythroid differentiation when ectopically expressed in myeloid leukemic K562 cells (Figure 8). This is consistent with the role of its transcriptional activator, KLF1, in driving terminal erythroid differentiation [44]. From this study, we cannot discount the possibility that the chimeric transcripts driven by KLF1 may be biologically functional in some circumstances. Indeed, host exaptation of TEs by exonization or through the remodelling of expression programs is a phenomenon of which multiple instances have been described [5,13,14,19]. Incidentally, although the consensus sequence of the *ORR1A0* exon contains a number of short open reading frames (see Figure 2B), each ATG is ultimately succeeded by an in-frame stop codon. Thus *ORR1A0* promoters are predicted to drive expression of either full-length endogenous proteins (where a canonical translation start site lies in a downstream, spliced exon), or N-terminal truncated protein isoforms, in cases where internal ATGs are suitable start sites of translation, as for PU.2.

The importance of appropriate retrotransposon control is apparent from numerous examples in which dysregulation results in disease [25,26]. It is unclear as to the extent to which the dysregulation of *ORR1A0* transcription affects the physiology of the *Klf3* null mice. However, these animals do display an erythroid phenotype, with impaired maturation, reticulocytosis, increased Howell-Jolly bodies and decreased lifespan of erythrocytes [49]. These defects have not been attributed to any single gene and it is more likely that they arise as a complicated result of multiple defects including the widespread expression of aberrant, chimeric transcripts that we have presented here.

## Conclusions

In summary, these findings emphasize the non-redundant role that KLF3 plays in preventing widespread, promiscuous transcription specifically from the *ORR1A0* LTR. We suggest that KLF3 is likely one of a patchwork of zinc finger proteins including ZFP809 that together collaborate to silence the diverse collection of TEs that constitute such a large fraction of vertebrate genomes.

## Materials and methods

### *Klf3*^−/−^ mice

Generation and maintenance of the *Klf3*^−/−^ mouse line has been described previously [66]. Ethical approval for animal use was obtained from the appropriate Animal Care and Ethics Committees (University of Sydney, approval number L02/7-2009/3/5079; and University of New South Wales, approval number 09/128A). Genomic DNA was extracted from tail snips using DirectPCR Lysis Reagent (Viagen Biotech Inc, Los Angeles, CA, USA) as per the manufacturer’s instructions and genotyping was performed as described previously [66].

### Sorting of TER119^+^ fetal liver cells

TER119^+^ cells were sorted from whole fetal livers (E13.5 or E14.5) using anti-TER119 Microbeads with MS or LS columns (Miltenyi Biotec Australia, North Ryde, NSW, Australia) as per the manufacturer’s protocol. After eluting, cells were centrifuged at 300 g for 10 min at 4°C and RNA was extracted from the cell pellet as described below.

### RNA extraction and quantitative real-time RT-PCR

RNA was extracted, purified and subjected to DNase treatment as described previously [45,49]. Extracted RNA was then used as a template for cDNA synthesis using the SuperScript^®^ VILO™ cDNA Synthesis Kit (Invitrogen, Carlsbad, CA, USA) as instructed by the supplier. Quantitative real-time RT-PCR reactions were set up as described previously [45,49] but using FastStart Universal SYBR Green Master (ROX) (Roche Diagnostics Australia Pty Ltd, Castle Hill, NSW, Australia) or *Power* SYBR^®^ Green PCR Master Mix (Life Technologies, Gladesville, NSW, Australia). Reactions were run using the Applied Biosystems 7500 Fast Real-Time PCR System (Life Technologies) and data were analyzed using 7500 Software v2.0.4 (Life Technologies). Relative quantification was calculated using *18S* rRNA levels and standard curves derived from serial dilutions of amplicon as described previously [45].

### Real-time RT-PCR primers

Gene-specific primer pairs were designed using the Primer-BLAST tool [67] or PrimerExpress v3.0 (Applied Biosystems, Foster City, CA, USA) software to cross exon boundaries where possible. Primers were included in reactions at a concentration of 400 nM. The sequences of forward and reverse primers for each gene are: *18S*, 5′-CACGGCCGGTACAGTGAAAC-3′ and 5′-AGAGGAGCGAGCGACCAA-3′; *Pu.1* exons 2/3, 5′-CCTTCGTGGGCAGCGATGGA-3′ and 5′-GAGCTGCTGTAGCTGCGGGG-3′; *Pu.1* exons 3/4, 5′-GAGCTGGAACAGATGCACGTCCT-3′ and 5′-GTGGGCTGGGGACAAGGTTTGAT-3′; *Pu.1* exons 4/5, 5′-TGGAGAAGCTGATGGCTTGGAGC-3′ and 5′-CAGCAGGAACTGGTACAGGCGAA-3′; *Pu.2* exons 2b/3, 5′-CCTGGATGCTGTCATGCTCCCAA-3′ and 5′-GTTGTTGTGGACATGGTGTGCGG-3′; *Znrf2*, 5′-CCACTAGCAGCCTTTGGATGAAGACA-3′ and 5′-GGGTACAAATTTTGAGCATACCGGGC-3′; *Brca2*, 5′-CTGCCATGCTCCCACCTTGATGA-3′ and 5′-ACCGTGGGGCTTATACTCAGATTCCT-3′; *Pqlc3*, 5′-ACTCTCAGCTCTGCCTGTGCCAT-3′ and 5′-GGTGAGCAGTGGGTTCCCATAGT-3′; *Bzw2*, 5′-GCCATGCCTGCCTGGATA-3′ and 5′-ACTGGCTTCTGATGCTTATTCATAAA-3′; *Cpe*, 5′-GCTCCTCCTGTGCCATGCT-3′ and 5′-CATGCATGTTCCCAATGTATTTAAA-3′; *Cd59b*, 5′-GCTCCCACCTTGATGATAATGG-3′ and 5′-TGAGTCCCCTCTGAGCTCTCA-3′; *Tmx4*, 5′-CCACCTTGATGATAATGGACTGAA-3′ and 5′-TGCTGGGAGAGTGGTGACAA-3′; *Tcfl5*, 5′-GCCATGCCTGCCTGGAT-3′ and 5′-TGGATGTCGAATAAGAGTTACCAAAAG-3′; *HBZ*, 5′-GAGGACCATCATTGTGTCCA-3′ and 5′-AGTGCGGGAAGTAGGTCTTG-3′; *HBD*, 5′-AACCTCAAGGGCACTTTTTCT-3′ and 5′-GGAAACAGTCCAGGATCTCAA-3′; *ALAS2*, 5′-TAAGGCAACAAAGGCTGGAG-3′ and 5′-GCCTTCACATCTTCCTGGAC-3′; and *ERMAP*, 5′-GCTGTCTGTGCATGTGTCAG-3′ and 5′-CCACCTCACCTCCTTGGGTA-3′.

### 5′ RACE (Rapid Amplification of cDNA Ends)

600 ng total RNA from TER119^+^ fetal liver cells obtained from *Klf3*^+/+^ and *Klf3*^−/−^ embryos (E13.5) was used as a template for first strand cDNA synthesis using the SMARTer™ RACE cDNA Amplification Kit (Clontech, Mountain View, CA, USA). The RACE PCR was performed as directed by the supplier but using 0.05 unit/μL REDTaq DNA Polymerase (Sigma Aldrich, St Louis, MO, USA). Thermal cycler settings were 24 cycles of 94°C/30 s, 64°C/30 s, and 72°C/1 min. Amplified products were resolved by electrophoresis through a 1.2% agarose gel and were purified using the Wizard SV Gel and PCR Clean-up System (Promega Corporation, Madison, WI, USA) as per the manufacturer’s manual. Nested RACE PCR was then performed as described above with slight alterations to the thermal cycler parameters: 24 cycles of 94°C/30 s, 60°C/30 s, and 72°C/1 min. First round and nested RACE PCR primers are listed respectively: reverse primer targeting exon 4 of *Pu.1*, 5′-GTGGGCTGGGGACAAGGTTTGAT-3′; reverse primer targeting exon 3 of *Pu.1*, 5′- GCTGTAGCTGCGGGGGCTGCACACT-3′. Amplicons were resolved and purified as described above and were sequenced by the Australian Genome Research Facility Ltd, Brisbane, QLD, Australia.

### Chromatin immunoprecipitation (ChIP)

ChIP was performed with slight modifications based on Schmidt *et al*. [68]. Briefly, one cross-linked E14.5 liver was used per IP with antibodies as follows: IgG, 5 μg sc-2027 (Santa Cruz Biotechnology, Santa Cruz, CA, USA); anti-H3K4me3, 5 μg C42D8 (Cell Signaling Technology, Danvers, MA, USA), or 5 μg ab12209 (abcam, Cambridge, MA, USA); anti-H3K9me3, 5 μg ab8898 (abcam); anti-H3K27me3, 3 μg C36B11 (Cell Signaling Technology) or 5 μg ab6002 (abcam); anti-H3K27ac, 0.5 μg #4353 (Cell Signaling Technology) or 5 μg ab4729 (abcam). Real-time PCR quantification of chromatin pull-down was performed as described above and amounts were normalized to the level of input material prior to immunoprecipitation. ChIP primer sequences have been described previously for *Klf8* promoter 1a and *Fam132a*[49,50]. Other primers used are as follows: *Pu.2 ORR1A0* -250 bp, 5′-GAAGTCCTTCTGGCTTCTGCAT-3′ and 5′-CTGACCTTGTCTAACCCTTTTGTTTA-3′; *Pu.2 ORR1A0* -120 bp, 5′-GACAAGGTCAGGAGAGGTTT-3′ and 5′-CCACAGTGACACACCCAT-3′; *Myod1*, 5′-TCCTATGCTTTGCCTGGTCT-3′ and 5′-GGAAGGAGGGCAGAGAGACT-3′; *Serpina9*, 5′-TGTGCTGGACCTGGTTTGTA-3′ and 5′-CTGGCAGCTCTCACCTCTCT-3′, and; *Gapdh*, 5′-GACAGTCGGAAACTGGGAAG-3′ and 5′-CATCACGTCCTCCATCATCC-3′. Base positions refer to where amplicons are centered relative to the TSS.

### Vectors and cloning of PU.1 and PU.2

The vectors pPac and pPac-Klf1 were provided by Menie Merika and Stuart Orkin (Harvard Medical School, Boston, MA, USA). The plasmid pPac-Klf3 and the mammalian expression vectors pMT3 and pMT3-Klf3 were gifts from Dr José Perdomo (St George Clinical School, Sydney, Australia). The mammalian expression vector pSG5-Klf1 was supplied by James Bieker (Mount Sinai School of Medicine, New York, NY, USA). *Firefly* and *Renilla* luciferase vectors used were pGL4.10[*luc2*], and pGL4.23[*luc2*/minP] and pGL4.74[*hRluc*/TK] as transfection controls (Promega Corporation). The vector pEF-IRES-puro5 (pEF1α) was kindly provided by Dr Daniel Peet (University of Adelaide, Adelaide, Australia). The *Renilla* luciferase reporter construct pLightSwitch-Clec5a, containing approximately 1.1 kb of the human *CLEC5A* promoter, was purchased from Switchgear Genomics (Menlo Park, CA, USA). The *Pu.2 ORR1A0* promoter (−140 to +23) was synthesized by GeneArt^®^ (Life Technologies) and subcloned into *Kpn*I/*Xho*I pGL4.10[*luc2*] to create pGL4.10-*ORR1A0*.

Full length *Pu.1* and *Pu.2* were cloned from the cDNA generated during the 5ߣĻ߰ RACE described above. PCRs were set up using Phusion High-Fidelity DNA Polymerase (Finnzymes OY, Espoo, Finland) as directed by the supplier. Primers used in the first round of amplification include: 10x Universal Primer A Mix (UPM) supplied from SMARTer™ RACE cDNA Amplification Kit (Clontech) as a forward primer, and a reverse primer specific for exon 5 of *Pu.1*; 5′-TCCGGGCCGGGCGACGGGTTAATGCTAT-3′. Thermal cycler parameters were 98°C/30 s, followed by 25 cycles of 98°C/10 s, 59°C/30 s, and 72°C/1 min, and a final cycle of 72°C/5 min. Amplified *Pu.1* and *Pu.2* products were resolved by electrophoresis, purified as described above and subjected to nested PCR using foward primers covering the start points of translation for PU.1 (5′-ATTACTCGAGGCTCAGCTGGATGTTACAGGCGTGCAAA-3′) and PU.2 (5′-ATTACTCGAGGCCACCATGGAGCTGGAACAGATGCAC-3′) together with the common reverse primer 5′-TAATGAATTCAGCCTGGCGGTCTCTGCGGGCGATCAGT-3′ (which includes the *Pu.1* stop codon in exon 5). Parameter settings were 98°C/30 s, followed by 25 cycles of 98°C/10 s, 69°C/30 s, and 72°C/1 min, and a final cycle of 72°C/5 min. The fragments were subsequently cloned into *Xho*I/*Eco*RI pMT3 to form pMT3-Pu.1 and pMT3-Pu.2, respectively. *Pu.1* and *Pu.2* were then subcloned into *Xho*I/*Eco*RI pEF1α to generate pEF1α-Pu.1 and pEF1α-Pu.2. Similarly, *Pu.1* and *Pu.2* were cloned into *BglII/HpaI* pMSCVpuro and pMSCVhyg (Clontech), respectively, using the forward primers 5′-ATTAAGATCTGCTCAGCTGGATGTTACAGGCGTGCAAA-3′ and 5′- ATTAAGATCTGCCACCATGGAGCTGGAACAGATGCAC -3′ and the reverse primer 5′-TAATGTTAACAGCCTGGCGGTCTCTGCGGGCGATCAGT-3′.

### Cell culture

COS cells were cultured as described previously [45]. K562 and HL60 cells were maintained similarly but in RPMI 1640 culture medium (Gibco-BRL Life Technologies, Grand Island, NY, USA). Culture conditions for B1.6 erythroblast cells have been described elsewhere [56]. B1.6 cells were induced with tamoxifen as described previously [45] and, with the exception of the time-course and cycloheximide experiments, were harvested for RNA or nuclear extracts after 48 h. SL2 cells were cultured in Schneider’s Drosophila medium (Gibco-BRL Life Technologies, Grand Island, NY, USA) supplemented with 10% heat-inactivated FCS and 1% penicillin/streptomycin/glutamine solution at 24°C.

### Transfections and retroviral infections

COS cells were transfected with 5 μg pMT3-Pu.1, pMT3-Pu.2 or pMT3 empty using FuGENE6 (Roche Diagnostics) as instructed by the supplier. Cells were harvested after 48 h for nuclear extracts. For retroviral infection of K562 cells, Phoenix A packaging cells were transfected with 12 μg total vector DNA (12 μg pMSCVpuro-Pu.1, 12 μg pMSCVhyg-Pu.2, 6 μg pMSCVpuro plus 6 μg pMSCVhyg, or 6 μg pMSCVpuro-Pu.1 plus 6 μg pMSCVhyg-Pu.2) using Lipofectamine 2000 (Life Technologies) according to the manufacturer’s instructions. Target K562 cells were seeded at 1 × 10^5^ cells/mL in 6-well Plates 24 h prior to infection. Forty-eight hours following infection of packaging cells, virus-containing media (VCM) were collected and passed through a 0.45 μm low protein binding filter. Non-tissue culture treated 6-well dishes were then coated with RetroNectin^®^ (Clontech) as instructed by the manufacturer. Half the volume of VCM was then added to the 6-well dishes for 30 min at room temperature. The target K562 cells were resuspended in the remaining VCM with the addition of protamine to a final concentration of 8 μg/mL. After 30 min, the VCM was aspirated from the coated wells and was seeded with the K562 cells. The plates were subsequently centrifuged at 400 g for 1.5 h at 30°C and then incubated at 37°C with 5% CO_2_ overnight. After 12 h, VCM was collected from the packaging cells for a second round of infection as described above. Eight hours after the second spinoculation, the K562 cells were replenished with additional VCM and incubated for 48 h before replating in RPMI 1640 culture medium with 10% (v/v) heat-inactivated FCS (Gibco) and 1% (v/v) penicillin/streptomycin/glutamine solution (Gibco). After another 48 h, cells were subjected to antibiotic selection and maintained in 1 μg/mL puromycin dihydrochloride (Sigma) and 200 μg/mL hygromycin B (Life Technologies) as appropriate.

Separately, K562 cells were also transfected with pEF1α-Pu.2 or pEF1α empty using the Gene Pulser Xcell electroporation system (Bio-Rad, Hercules, CA, USA). Briefly, 10^6^ cells and 20 μg plasmid were resuspended in Dulbecco’s modified eagle medium (DMEM) low glucose (LG) (Gibco-BRL Life Technologies) without serum and in a total volume of 400 μL. Cells were electroporated at 200 V, 950 μF and subsequently cultured and maintained in 2 μg/mL puromycin dihydrochloride to generate monoclonal lines.

### Nuclear extracts and electrophoretic mobility shift assays (EMSAs)

Nuclear extracts were obtained and EMSAs were performed as described previously [46]. The radiolabelled probe containing the PU.1 consensus binding site comprises sense and antisense oligonucleotides for the sequence 5′-GCTCGAGGACTTCCTCTTTCCAGTGC-3′ as described elsewhere [69]. *ORR1A0* double stranded CACCC probe sequences are as follows: CACCC#1, 5′-ATGGTGCCACTCCCTGGTCC-3′; CACCC#2, 5′-CAGTGACACACCCATTCCA-3′; CACCC#3, 5′-TTATACCCACACCCACAGTG-3′; CACCC#4, 5′-TAAGATCCTCACCCTAGTTG-3′. The positive control KLF3 binding site in the *Fam132a* promoter is ‘Probe C’ from [54]. The antibodies used that were specific for the N-terminus and C-terminus of Pu.1 were 9G7 (Cell Signaling Technology) and T-21 (Santa Cruz Biotechnology), respectively. KLF1- and KLF3-specific antisera have been described previously [46].

### Western blotting

Western blots were performed by standard methods. Briefly, nuclear extracts were separated by SDS-PAGE and were electrotransferred to PVDF membrane, which was then blocked with 5% skim milk in 50 mM Tris–HCl (pH 7.4), 150 mM NaCl, and 0.05% Tween 20. PU.2 protein was probed by overnight incubation of membrane in 5% skim milk with 0.2 μg/mL PU.1 antibody (T-21) (Santa Cruz Biotechnology) at 4°C. Detection was achieved using Immobilon Western Chemiluminescent HRP Substrate (Millipore Corporation, Billerica, MA, USA) and subsequently, membranes were stripped in 0.2 M NaOH for 10 min and were probed with β-actin antibody (Sigma).

### Reporter assays

SL2 cells were split into 6-well plates at a concentration of 5 × 10_5_/mL and 24 h later were transfected with pPac-Klf1 (0, 50, or 250 ng) and pPac-Klf3 (0, 12.5, 25, 50, or 100 ng) supplemented to equal loads with pPac empty vector, together with 100 ng pGL4.74 [*hRLuc*/TK] and 1 μg pGL4.10 [*luc2*] or pGL4.10-ORR1A0 using FuGene6 (Roche Diagnostics) as instructed by the supplier. In competition assays, the pGL4.10-ORR1A0 vector (and pGL4.10 [*luc2*]) were driven by co-transfection of 200 ng pPac-Klf1. After 48 h, cells were harvested and lysates analyzed using the Dual-luciferase^®^ Reporter Assay System (Promega Corporation) and a TD20/20 luminometer (Turner Biosystems, Sunnyvale, CA, USA). HEK293 cells were similarly transfected but with 0, 10, 100, and 1,000 ng pEF1α-Pu.1 (supplemented with pEF1α to a total of 1,000 ng vector) together with 1 μg pLightSwitch (empty vector) or pLightSwitch-Clec5a. As a control, 100 ng pGL4.23 [*luc2*/minP] *Firefly* luciferase vector was co-transfected. In competition assays, HEK293 cells were transfected and analyzed as above but with 1 μg pEF1α-Pu.1 together with 0, 10, 100 and 1,000 ng pEF1α-Pu.2.

### Microarrays

Microarray data from TER119^+^ E14.5 fetal liver cells from *Klf3*^+/+^ and *Klf3*^−/−^ embryos have previously been described [49]. Monoclonal K562 cell lines stably transfected with pEF1α or pEF1α-Pu.2 (*n* = 3 each) were harvested for total RNA which was subsequently hybridized to Affymetrix Human Gene 1.0 ST arrays according to the manufacturer’s instructions (Affymetrix, Santa Clara, CA, USA). Hybridization and processing were performed by the Ramaciotti Centre for Gene Function Analysis (University of New South Wales, Sydney, Australia). Data were analyzed using Affymetrix^®^ Expression Console™ software (Affymetrix). Microarray data are available in the Gene Expression Omnibus database [70] under accession number GSE50083.

### High throughput RNA-sequencing (RNA-Seq)

RNA was extracted from TER119^+^-sorted liver cells from three *Klf3*^+/+^ and three *Klf3*^−/−^ litter-matched E14.5 embryos (two litters total). Libraries were prepared using 1 μg total RNA using the TruSeq RNA Sample Prep Kit v2 (Illumina, San Diego, CA, USA) according to the manufacturer’s instructions. The six libraries were multiplexed into two lanes using sample specific adapters such that there were three samples per lane. 100 bp paired end reads were sequenced using TruSeq v3 SBS reagents on the Hiseq 2000 (Illumina, San Diego, CA, USA). Library preparation and sequencing were performed by the Ramaciotti Centre, University of New South Wales, New South Wales, Australia. Quality control on the reads was performed using FastQC v0.10.1 available from [71].

Two separate strategies were used for alignment and RNA-seq analysis. The first of these sought to uniquely map reads from repeat elements by using high stringency alignment cut-offs and was primarily employed to visualize chimeric splicing events using Integrative Genomics Viewer [72]. Reads were aligned to the mm10 *Mus musculus* genome using tophat2 (v2.0.4) using the default settings except for the following (-r -40 --segment-length 50 --coverage-search --segment-mismatches 0 -g 50 --genome-read-mismatches 0 --read-mismatches 0 -I 200000 --no-discordant --no-mixed --b2-L 30 --b2-D 10000 --b2-R 100 -n 0 -M) [73]. The second approach used more relaxed cutoffs to better measure expression levels of non-repeat exons. This latter approach was employed to determine differential gene and isoform expression (Additional file 4: Tables S1 and Additional file 5: Table S2). Again, reads were aligned to the mm10 *Mus musculus* genome using the default settings of tophat2 (v2.0.4), except for the following (--no-discordant -M --no-coverage-search --microexon-search -n 3 --genome-read-mismatches 3 --read-mismatches 3 --b2-sensitive -G) [73]. Transcripts were assembled using Cufflinks v2.0.2 and the mm10 annotations were included [74]. Transcripts across all replicates were merged using cuffmerge and differential expression analysis was performed pair-wise on the groups using cuffdiff. A q-value (FDR) threshold of <0.05 was used to determine significantly differentially expressed genes. RNA-Seq data have been deposited in the Gene Expression Omnibus under the accession number GSE50554.

### Bioinformatics

The sequence and genomic positions of *ORR1A0* LTR elements were determined using the RepeatMasker program [75] in conjunction with the University of California Santa Cruz (UCSC) Genome Browser [76]. Sequence information of retroelement families was obtained using Repbase Update [57,77]. The overlap between differentially expressed loci as measured by RNA-Seq and *ORR1A0* and *ORR1A0*-*int* elements was established using the intersect command in Bedtools v2.17.0 [78]. Similarly, overlaps (using 100 bp windows) were determined between *ORR1A0* elements and: TRIM28 ChIP peaks (from [40,64]); TRIM28-dependent H3K9me3 peaks (from [40]), and regions of DNA methylation in hematopoietic stem cells and erythroblasts (from [65]). Gene Expression Omnibus accession numbers for the data sets analyzed are GSM1032198, GSM773067, GSM1032190, and GSE38354, respectively.

### Additional files

#### Description of additional files

The following additional data are available with the online version of this paper. Additional file 1: Figure S1 shows that the chimeric *Pu.1* transcript (*Pu.2*) is predominantly upregulated in erythroid tissues in the absence of KLF3. Additional file 2: Figure S2 shows levels of H3K9 tri-methylation at the *Pu.2* promoter in *Klf3*^−/−^ and wild-type E14.5 fetal liver cells. Additional file 3: Figure S3 shows that *Pu.2* and other *ORR1A0* chimeric transcripts are rapidly activated by KLF1 and in the presence of cycloheximide, suggesting that they are direct targets. Additional file 6: Figure S4 provides validation of genes to which the *ORR1A0* exon is spliced in *Klf3*^−/−^ E14.5 TER119^+^ fetal liver cells. Additional file 7: Figure S5 gives further examples of de-repressed *ORR1A0* transcripts in the absence of KLF3. Additional file 8: Figure S6 contains reporter assay data demonstrating the opposing transcriptional activities of PU.1 and PU.2. Additional file 4: Table S1 shows the list of genes that are significantly, differentially expressed in *Klf3*^−/−^ TER119^+^ fetal liver cells by RNA-Seq. Additional file 5: Table S2 shows the list of genes that have significantly de-repressed isoforms. Additional file 9: Table S3 shows microarray results from K562 cell stably expressing PU.2.

## Competing interests

The authors declare that they have no competing interests.

## Authors’ contributions

KSM, APWF, JB, and LJN performed experiments and analyzed data. Specifically, APWF, KSM, and LJN performed cell sorting experiments and qRT-PCR. KSM conducted 5′ RACE, histone ChIPs, reporter assays, western blots, and microarrays. APWF and KSM carried out EMSA experiments and generated the stable K562 lines. APWF performed B1.6 cell work. APWF and JB designed, performed and analyzed the RNA-Seq and conducted bioinformatics comparisons. APWF, MC, and RCMP contributed to overall design of the study and directed research. APWF, KSM, JB, MC, and RCMP wrote the paper. All authors read and approved the final manuscript.

## Supplementary Material

Additional file 1: Figure S1 The chimeric *Pu.1* transcript (*Pu.2*) is predominantly upregulated in erythroid tissues in the absence of KLF3. Total RNA was extracted from adult tissue from three *Klf3*^+/+^ (WT) and three *Klf3*^−/−^ (KO) mice and analyzed by quantitative real-time RT-PCR using primers specific for the exon 2b/3 junction **(A)** or exon 2/3 junction **(B)** of *Pu.1*. Levels have been normalized to *18S* rRNA and the lowest detectable reading in each chart has been set to 1.0. Error bars represent standard error of the mean. *, *P* <0.05 (Student’s two-tailed *t-*test) compared to wild-type. Click here for file

Additional file 2: Figure S2 The relative enrichment of H3K9me3 at the *Pu.2* promoter in *Klf3*^+/+^ and *Klf3*^−/−^ E14.5 fetal liver cells. ChIP data have been expressed as percentage input for each locus (*n =* 2 for each IP for *Klf3*^+/+^ (WT) or *Klf3*^−/−^ (Klf3 KO)). Error bars represent standard error of the mean. The *Pu.2* promoter shows a moderate level of H3K9 tri-methylation relative to positive (*Serpina9*) and negative *(Gapdh*) control loci. Click here for file

Additional file 3: Figure S3 *Pu.2* and *ORR1A0* chimeric transcripts are induced rapidly by KLF1-ER and in the presence of cycloheximide. RNA from KLF1-ER inducible B1.6 erythroblast cells was analyzed by qRT-PCR using primers specific for *Pu.2***(A, B)**, *ORR1A0-Znrf2***(C, D)**, and *ORR1A0-Brca2***(E, F)**. In (A, C, and E), RNA was harvested at 0, 2, 4, 8, and 24 h following tamoxifen treatment (*n* = 2). In (B, D, and F), cells were treated with cycloheximide for 30 min prior to tamoxifen addition (or ethanol for untreated), with samples being taken 8 h thereafter (*n* = 4 for each condition). All values have been normalized to *18S* rRNA levels and t = 0 h time points (A, C, and E) and tamoxifen-untreated samples (B, D, and F) have been set to 1.0. Error bars represent standard error of the mean. *, *P <*0.05 (Student’s one-tailed *t*-test) compared to t = 0 h (A, C, and E) and tamoxifen-untreated (B, D, and F). N.S, not significant. Click here for file

Additional file 4: Table S1 Genes that are significantly, differentially expressed in *Klf3*^−/−^ TER119^+^ fetal liver cells compared to *Klf3*^+/+^. RNA-Seq was performed on triplicate samples and differentially expressed genes were determined using a FDR cutoff of 0.05. Click here for file

Additional file 5: Table S2 The list of annotated genes which have significantly de-repressed isoforms (>10-fold) in *Klf3*^−/−^ TER119^+^ fetal liver cells compared to *Klf3*^+/+^. Eighty eight differentially expressed isoforms (FDR <0.05) were determined, covering 70 different genes. Click here for file

Additional file 6: Figure S4 Confirmation of further *ORR1A0* splicing events in *Klf3*^−/−^ erythroid cells that were not detected by RNA-Seq analysis. RNA from *Klf3*^+/+^ (WT), *Klf3*^+/−^ (HET), and *Klf3*^−/−^ (KO) TER119^+^ E14.5 fetal liver cells were analyzed by real-time RT-PCR using forward primers specific for *ORR1A0* and reverse primers recognizing downstream exons of the *Cd59b***(A)**, *Tmx4***(B)**, and *Bzw2*, *Cpe*, and *Tcfl5***(C)** genes. (A and B) Values have been normalized to *18S* rRNA levels and WT samples have been set to 1.0. Error bars represent standard error of the mean (*n* = 2 WT, 3 HET, and 3 KO). *, *P <*0.05 (Student’s two-tailed *t*-test) compared to both *Klf3*^+/+^ and *Klf3*^+/−^. **(C)** For these genes, the spliced transcripts were below the level of detection in *Klf3*^+/+^ cells and thus could not be quantified. RT-PCR products were electrophoresed on a 3% agarose gel and stained with ethidium bromide. Click here for file

Additional file 7: Figure S5 Further examples of de-repressed *ORR1A0* transcripts in the absence of KLF3. As in Figure 6, tracks represent RNA-Seq reads and splicing events for *Klf3*^+/+^ (WT) and *Klf3*^−/−^ (KO) E14.5 TER119^+^ fetal liver cell samples. **(A, B)***ORR1A0* elements are transcribed and spliced to downstream exons of *Znrf2* (A) and *Brca2***(B)**, which in turn are expressed at a significantly higher level in KO cells. **(C)** Spliced transcripts initiating nearby to an *ORR1A0* LTR are antisense to an annotated gene (*Dhx57*). **(D)** De-repressed transcription of an *ORR1A0*/*ORR1A0-int* cassette does not alter the expression of the surrounding gene (*Drosha*). Click here for file

Additional file 8: Figure S6 PU.2 opposes the transcriptional activity of PU.1. HEK293 cells were transfected with pLightSwitch *Renilla* luciferase reporter vector (promoter-less or containing the *CLEC5A* promoter). In **(A)**, increasing amounts of pEF1α-Pu.1 have been co-transfected, while in **(B)**, a steady amount of pEF1α-Pu.1 has been co-transfected together with increasing doses of pEF1α-Pu.2. In all experiments, pGL4.23[*luc2*/minP] *Firefly* luciferase vector was included as a transfection control and used for normalization. The means of triplicate experiments are shown and error bars represent standard error of the mean. *, *P* <0.05 (Student’s two-tailed *t*-test) compared to pLightSwitch-Clec5a wells transfected with 0 ng pEF1α-Pu.1 (A) or 0 ng pEF1α-Pu.2 (B). Click here for file

Additional file 9: Table S3 Erythroid genes are upregulated upon forced expression of PU.2 in K562 cells. Microarrays were performed on monoclonal K562 cell lines stably transfected with pEF1α-Pu.2 or pEF1α. A selection of erythroid genes that are upregulated >2-fold in cells expressing PU.2 is shown. Click here for file
