# Erratum to: Repression of chimeric transcripts emanating from endogenous retrotransposons by a sequence-specific transcription factor

**DOI:** 10.1186/s13059-016-0977-1

**Published:** 2016-06-03

**Authors:** Ka Sin Mak, Jon Burdach, Laura J. Norton, Richard CM Pearson, Merlin Crossley, Alister PW Funnell

**Affiliations:** School of Biotechnology and Biomolecular Sciences, University of New South Wales, Kensington, NSW 2052 Australia

In the study [1] a gel depicted in Fig. 2a was labelled in a way which suggests that the sample comes from a *Klf3*^−/−^ knockout mouse. In fact, this sample comes from a *Klf3*^−/−^*,Klf8*^genetrap^ double mutant animal. The *Klf8* genotype was not indicated as authors felt that it was not relevant for the conclusions of this paper; however, all authors now acknowledge that this information should have been included. Importantly, an equivalent result from single *Klf3*^−/−^ knockout mice is included and confirmed in the RNA-seq results presented in Figure 6 of the original article [1].

**Fig. 2:**
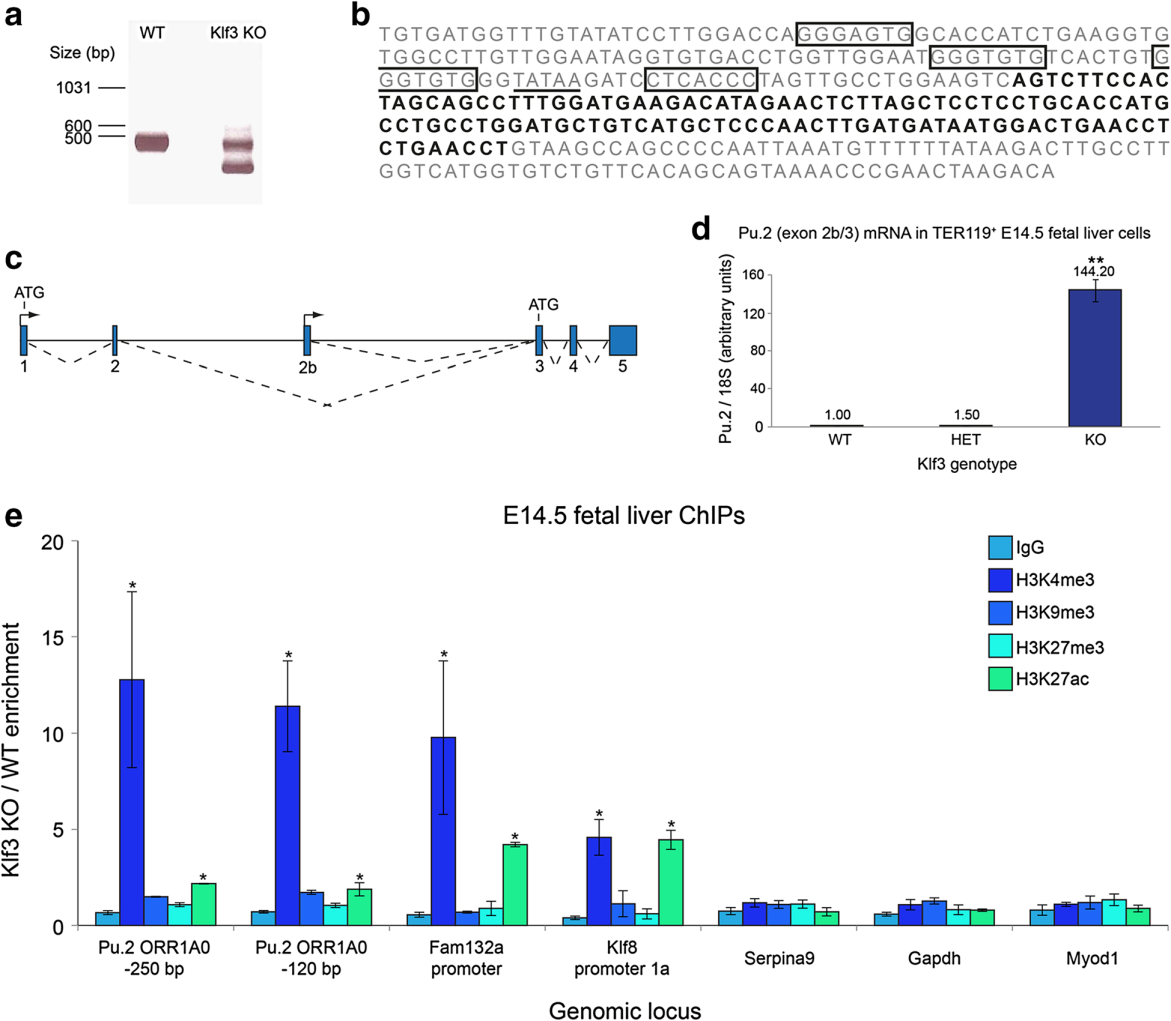
A novel, internal *Pu.1* promoter resides within an *ORR1A0* LTR element and is repressed by KLF3. (**a**) RNA from *Klf3* ^+/+^ (WT) and *Klf3* ^−/−^, *Klf8* ^genetrap^ TER119+ fetal liver cells was subjected to 5′ RACE using a reverse primer specific for exon 3 of *Pu.1* and analyzed by agarose gel electrophoresis. The smaller band from a *Klf3*KO animal was sequenced and found to contain a novel exon (exon 2b). (**b**) The sequence of the *ORR1A0* LTR, in which *Pu.1* exon 2b is shown in bold. Sequences which fit the KLF binding consensus 5′-NCN CNC CCN-3′ are boxed, and the TATA box at −30 is underlined. (**c**) Schematic of the murine *Pu.1* locus showing the position of exon 2b. Exons are represented by blue boxes, transcription start sites by arrowheads and splicing events by broken lines. Start points of translation (ATGs) for the two alternative transcripts are also shown. (**d**) Real-time RT-PCR quantification revealing that transcripts containing exon 2b spliced to exon 3 of *Pu.1* (that is, *Pu.2* transcripts) are upregulated in *Klf3* ^−/−^ TER119^+^ E14.5 fetal liver cells compared to *Klf3* ^+/−^ (HET) and *Klf3* ^+/+^. Values have been normalized to *18S* rRNA and the *Klf3* ^+/+^ sample has been set to 1.0. *n* = 3 for each genotype. **, P <0.005 compared to both *Klf3* ^+/+^ and *Klf3* ^+/−^ (Student’s two-tailedt-test). (**e**) ChIPs were performed on *Klf3* ^+/+^ and *Klf3* ^−/−^ E14.5 fetal livers (*n* = 2 or 3 of each genotype per IP). Data are represented as the fold-change enrichment in *Klf3* ^−/−^ cells compared to *Klf3* ^+/+^. The *Fam132a* and *Klf8* promoters have been included as positive controls while *Serpina9*, *Gapdh*, and *MyoD* are negative control regions. *, P <0.05 compared to *Gapdh* (Student’s one-tailed *t*-test). In (**d** and **e**), error bars represent standard error of the mean

All other data described in the article were obtained from the *Klf3*^−/−^ single knockout mice, and as such the conclusions of the article remain unchanged. It is also critical to note that all other results in the article could not have been obtained from the double mutant mice, because Klf3,Klf8 deficient animals die *in utero* (as reported by the authors in [2]).

Figure 2 with the correct legend is published in this Erratum.

The authors apologize for this omission and any confusion and inconvenience it may have caused.
