# Supplementary figures and images for: Repression of chimeric transcripts emanating from endogenous retrotransposons by a sequence-specific transcription factor
Source: Genome Biol. 2014 Apr 30;15(4):R58. doi: 10.1186/gb-2014-15-4-r58 (PMC4056533; doi:10.1186/gb-2014-15-4-r58)

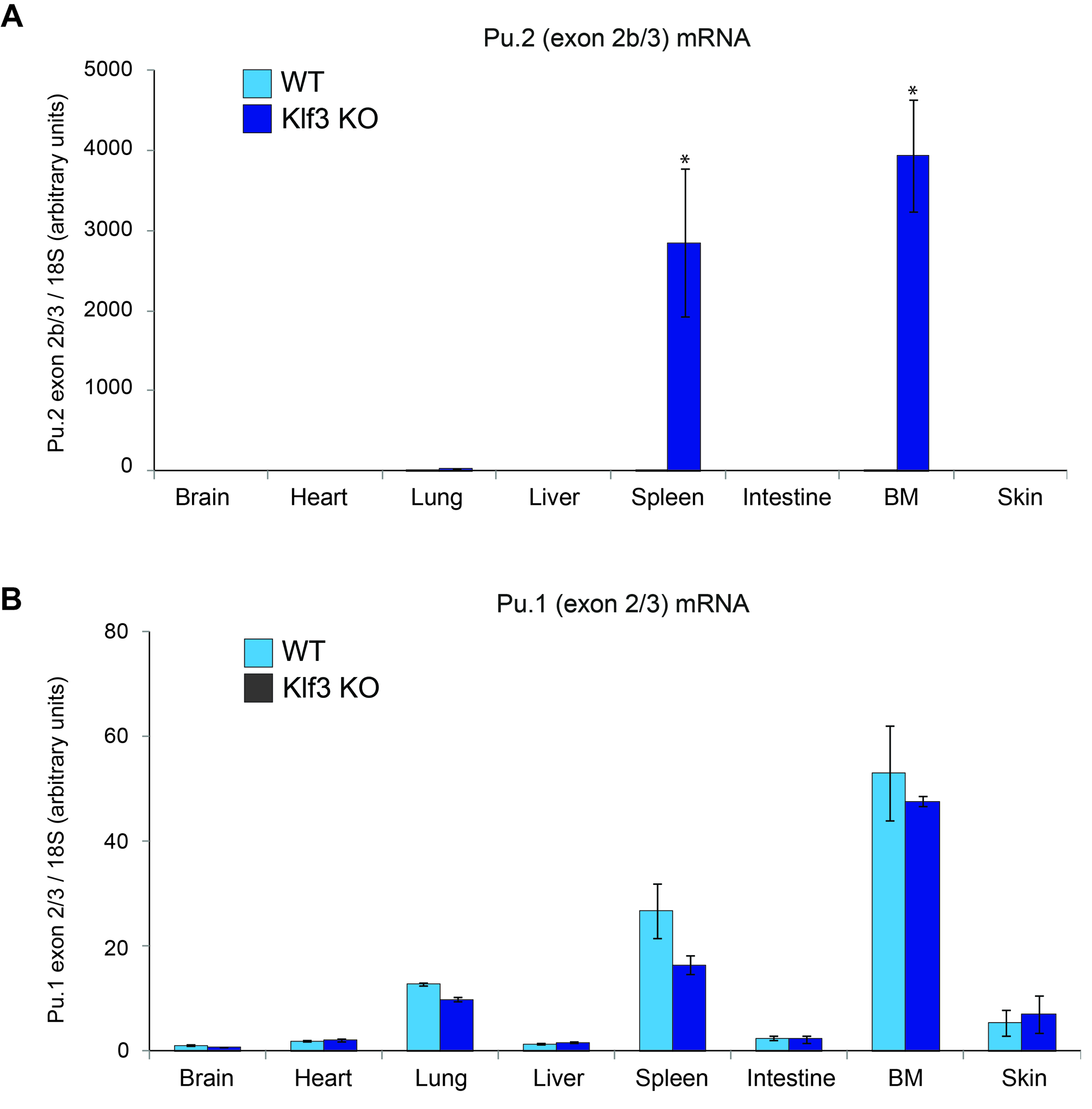

Supplement: Additional file 1: Figure S1 — The chimeric Pu.1 transcript (Pu.2) is predominantly upregulated in erythroid tissues in the absence of KLF3. Total RNA was extracted from adult tissue from three Klf3+/+ (WT) and three Klf3−/− (KO) mice and analyzed by quantitative real-time RT-PCR using primers specific for the exon 2b/3 junction (A) or exon 2/3 junction (B) of Pu.1. Levels have been normalized to 18S rRNA and the lowest detectable reading in each chart has been set to 1.0. Error bars represent standard error of the mean. *, P <0.05 (Student’s two-tailed t-test) compared to wild-type. [file gb-2014-15-4-r58-S1.tiff]

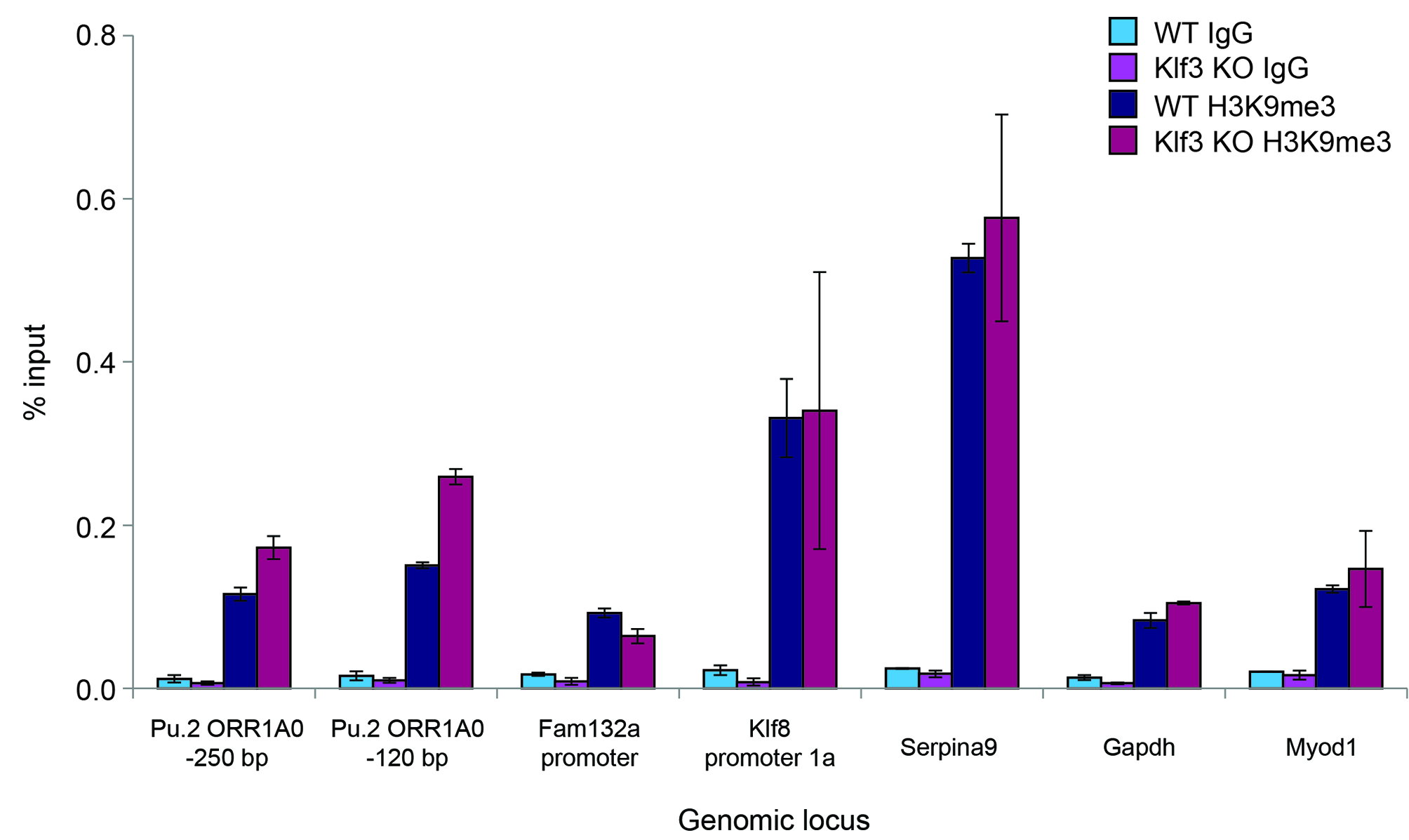

Supplement: Additional file 2: Figure S2 — The relative enrichment of H3K9me3 at the Pu.2 promoter in Klf3+/+ and Klf3−/− E14.5 fetal liver cells. ChIP data have been expressed as percentage input for each locus (n = 2 for each IP for Klf3+/+ (WT) or Klf3−/− (Klf3 KO)). Error bars represent standard error of the mean. The Pu.2 promoter shows a moderate level of H3K9 tri-methylation relative to positive (Serpina9) and negative (Gapdh) control loci. [file gb-2014-15-4-r58-S2.tiff]

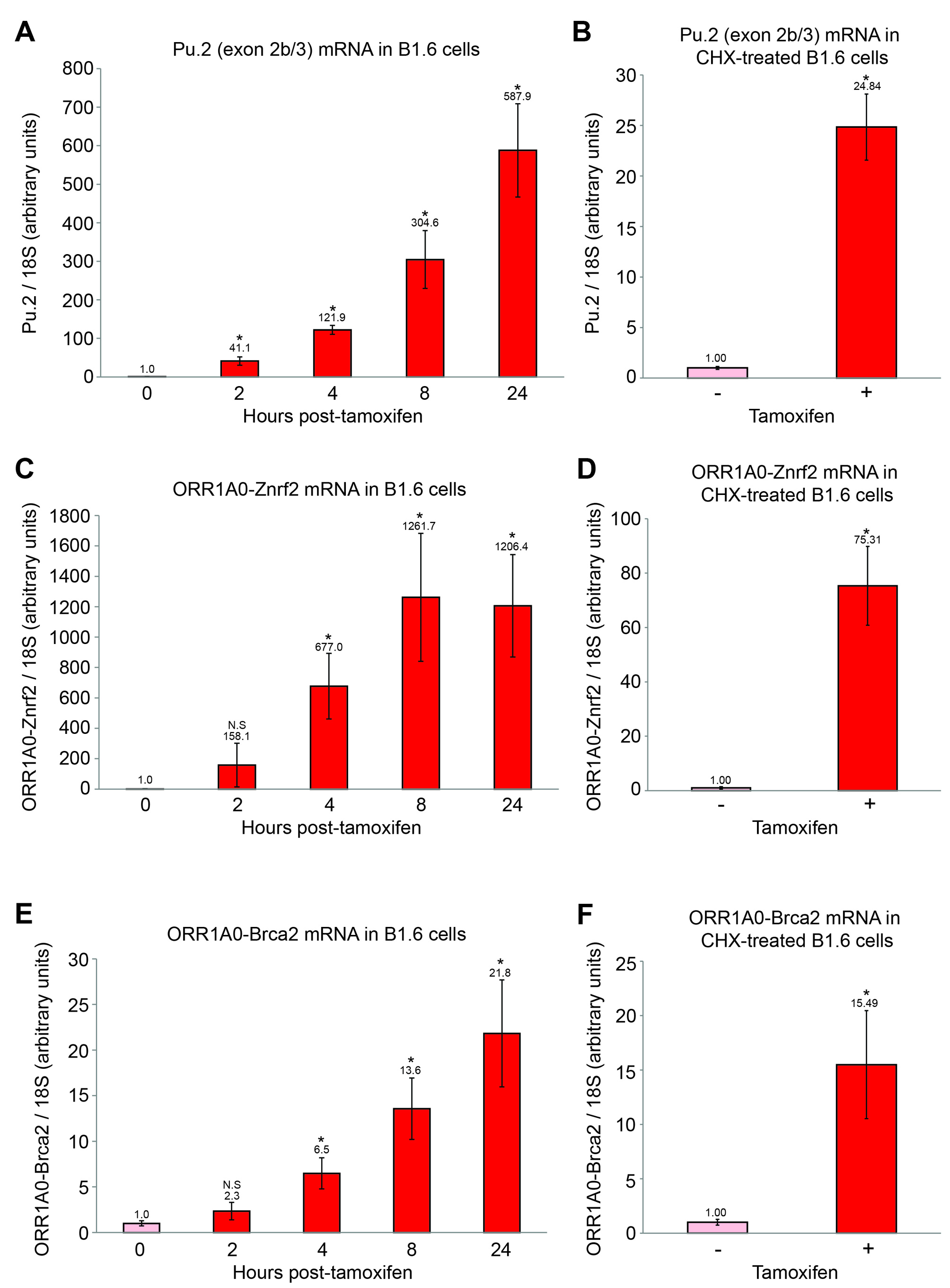

Supplement: Additional file 3: Figure S3 — Pu.2 and ORR1A0 chimeric transcripts are induced rapidly by KLF1-ER and in the presence of cycloheximide. RNA from KLF1-ER inducible B1.6 erythroblast cells was analyzed by qRT-PCR using primers specific for Pu.2(A, B), ORR1A0-Znrf2(C, D), and ORR1A0-Brca2(E, F). In (A, C, and E), RNA was harvested at 0, 2, 4, 8, and 24 h following tamoxifen treatment (n = 2). In (B, D, and F), cells were treated with cycloheximide for 30 min prior to tamoxifen addition (or ethanol for untreated), with samples being taken 8 h thereafter (n = 4 for each condition). All values have been normalized to 18S rRNA levels and t = 0 h time points (A, C, and E) and tamoxifen-untreated samples (B, D, and F) have been set to 1.0. Error bars represent standard error of the mean. *, P <0.05 (Student’s one-tailed t-test) compared to t = 0 h (A, C, and E) and tamoxifen-untreated (B, D, and F). N.S, not significant. [file gb-2014-15-4-r58-S3.tiff]

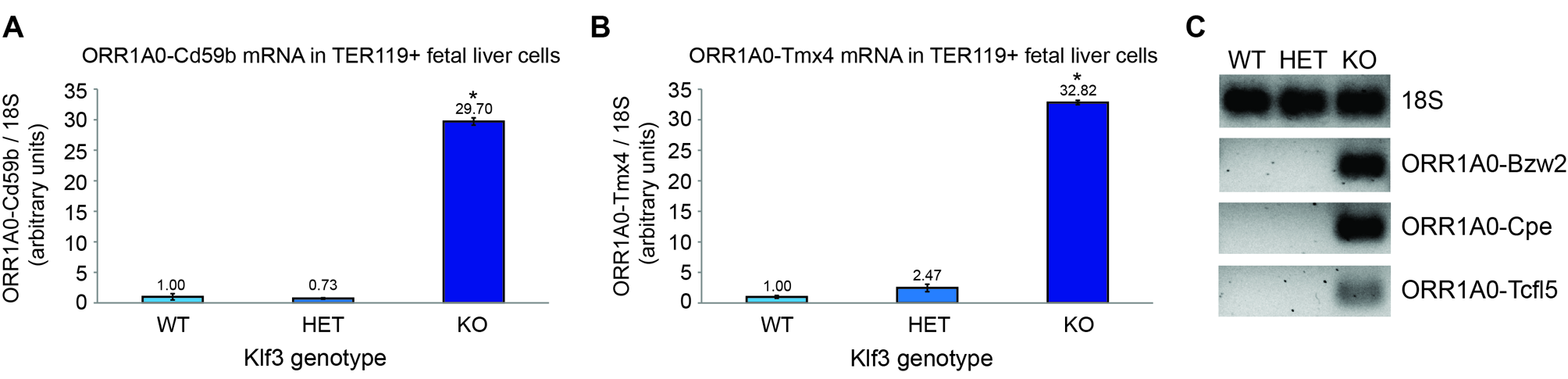

Supplement: Additional file 6: Figure S4 — Confirmation of further ORR1A0 splicing events in Klf3−/− erythroid cells that were not detected by RNA-Seq analysis. RNA from Klf3+/+ (WT), Klf3+/− (HET), and Klf3−/− (KO) TER119+ E14.5 fetal liver cells were analyzed by real-time RT-PCR using forward primers specific for ORR1A0 and reverse primers recognizing downstream exons of the Cd59b(A), Tmx4(B), and Bzw2, Cpe, and Tcfl5(C) genes. (A and B) Values have been normalized to 18S rRNA levels and WT samples have been set to 1.0. Error bars represent standard error of the mean (n = 2 WT, 3 HET, and 3 KO). *, P <0.05 (Student’s two-tailed t-test) compared to both Klf3+/+ and Klf3+/−. (C) For these genes, the spliced transcripts were below the level of detection in Klf3+/+ cells and thus could not be quantified. RT-PCR products were electrophoresed on a 3% agarose gel and stained with ethidium bromide. [file gb-2014-15-4-r58-S6.tiff]

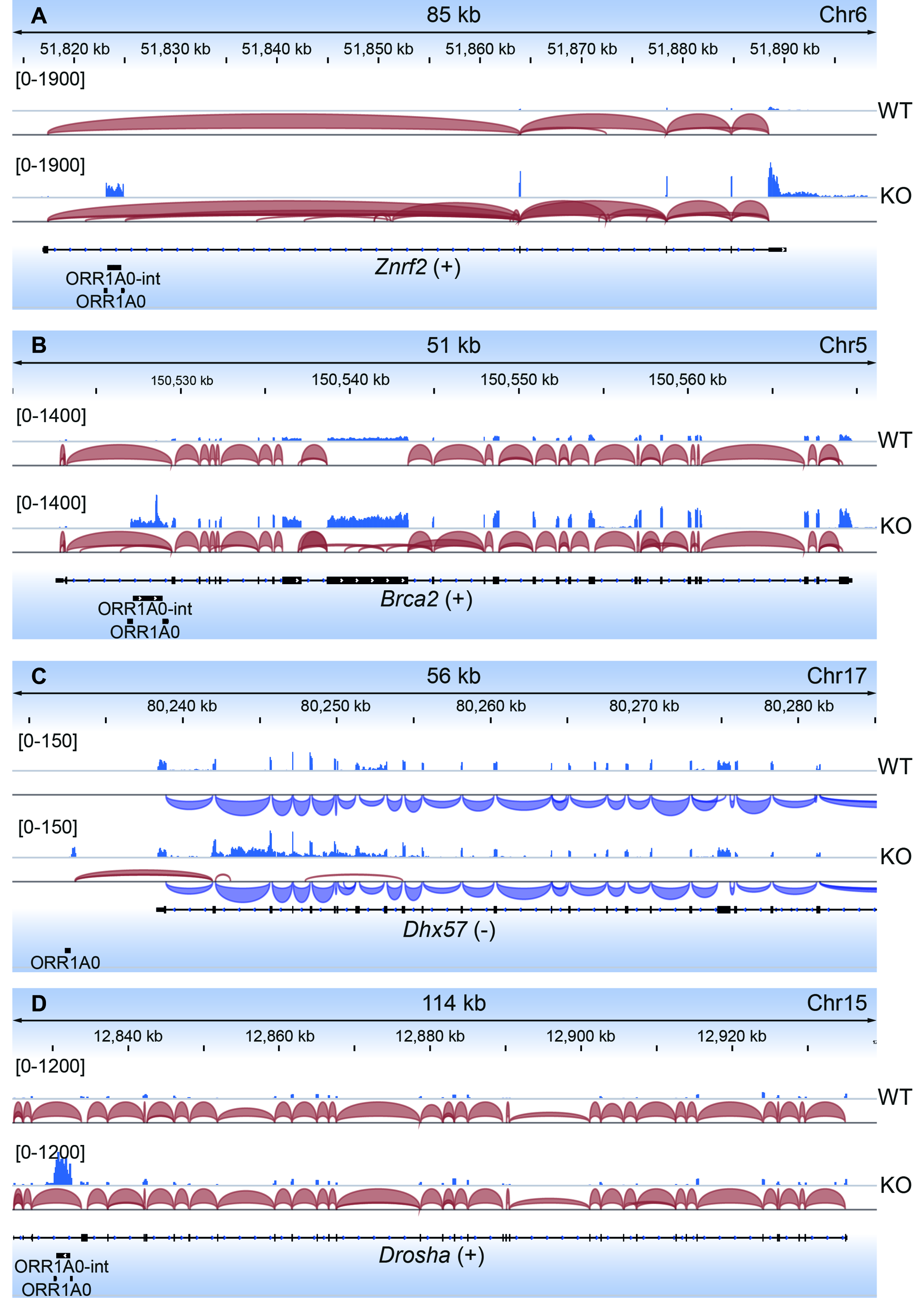

Supplement: Additional file 7: Figure S5 — Further examples of de-repressed ORR1A0 transcripts in the absence of KLF3. As in Figure 6, tracks represent RNA-Seq reads and splicing events for Klf3+/+ (WT) and Klf3−/− (KO) E14.5 TER119+ fetal liver cell samples. (A, B)ORR1A0 elements are transcribed and spliced to downstream exons of Znrf2 (A) and Brca2(B), which in turn are expressed at a significantly higher level in KO cells. (C) Spliced transcripts initiating nearby to an ORR1A0 LTR are antisense to an annotated gene (Dhx57). (D) De-repressed transcription of an ORR1A0/ORR1A0-int cassette does not alter the expression of the surrounding gene (Drosha). [file gb-2014-15-4-r58-S7.tiff]

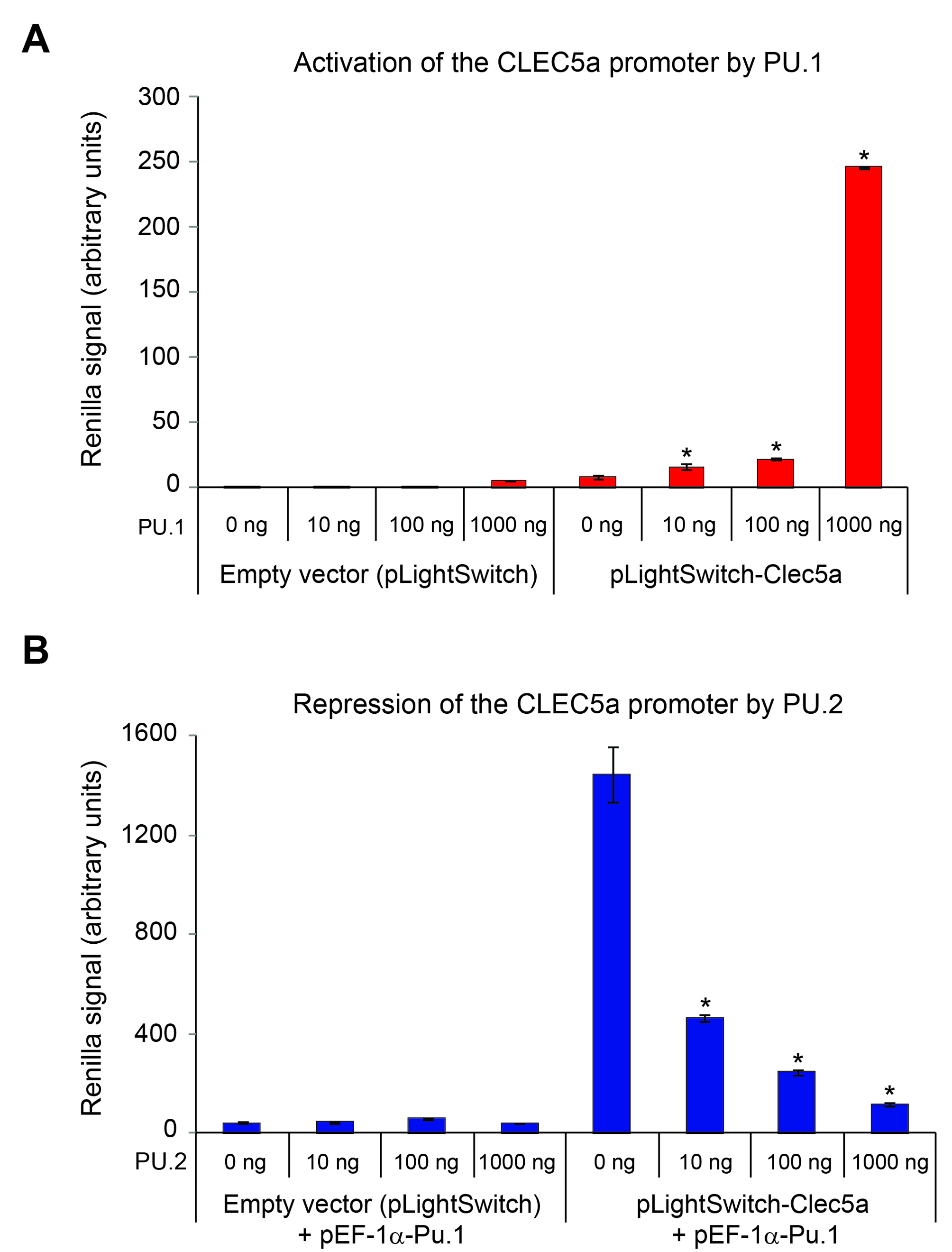

Supplement: Additional file 8: Figure S6 — PU.2 opposes the transcriptional activity of PU.1. HEK293 cells were transfected with pLightSwitch Renilla luciferase reporter vector (promoter-less or containing the CLEC5A promoter). In (A), increasing amounts of pEF1α-Pu.1 have been co-transfected, while in (B), a steady amount of pEF1α-Pu.1 has been co-transfected together with increasing doses of pEF1α-Pu.2. In all experiments, pGL4.23[luc2/minP] Firefly luciferase vector was included as a transfection control and used for normalization. The means of triplicate experiments are shown and error bars represent standard error of the mean. *, P <0.05 (Student’s two-tailed t-test) compared to pLightSwitch-Clec5a wells transfected with 0 ng pEF1α-Pu.1 (A) or 0 ng pEF1α-Pu.2 (B). [file gb-2014-15-4-r58-S8.tiff]
