# Supplementary material for: Repression of chimeric transcripts emanating from endogenous retrotransposons by a sequence-specific transcription factor
Source: Genome Biol. 2014 Apr 30;15(4):R58. doi: 10.1186/gb-2014-15-4-r58 (PMC4056533; doi:10.1186/gb-2014-15-4-r58)
Supplement: Additional file 9: Table S3 — Erythroid genes are upregulated upon forced expression of PU.2 in K562 cells. Microarrays were performed on monoclonal K562 cell lines stably transfected with pEF1α-Pu.2 or pEF1α. A selection of erythroid genes that are upregulated >2-fold in cells expressing PU.2 is shown. [file gb-2014-15-4-r58-S9.docx]

| Gene | pEF1α-Pu.2 / pEF1 α | Description |
| --- | --- | --- |
| ALAS2 | 8.07 | aminolevulinate, delta-, synthase 2, nuclear gene encoding mitochondrial protein, transcript variant 1, mRNA |
| HBD | 4.04 | hemoglobin, delta, mRNA. |
| CD36 | 3.96 | CD36 molecule (thrombospondin receptor), transcript variant 1, mRNA. |
| HBZ | 3.42 | hemoglobin, zeta (HBZ), mRNA. |
| GYPB | 3.08 | glycophorin B (MNS blood group), mRNA. |
| GYPE | 2.65 | glycophorin E (MNS blood group), transcript variant 2, mRNA. |
| HEMGN | 2.45 | hemogen, transcript variant 1, mRNA. |
| FECH | 2.26 | ferrochelatase (protoporphyria), nuclear gene encoding mitochondrial protein, transcript variant 1, mRNA. |
| RHCE | 2.24 | Rh blood group, CcEe antigens, transcript variant 1, mRNA. |
| KEL | 2.14 | Kell blood group, metallo-endopeptidase, mRNA. |
| HBA1 | 2.13 | hemoglobin, alpha 1, mRNA. |
| HBA2 | 2.13 | hemoglobin, alpha 2, mRNA. |
| RHD | 2.10 | Rh blood group, D antigen, transcript variant 1, mRNA. |
| GYPA | 2.01 | glycophorin A (MNS blood group), mRNA. |
| ERMAP | 2.01 | erythroblast membrane-associated protein (Scianna blood group), transcript variant 1, mRNA. |
